# Targeted delivery of ZNF416 siRNA-loaded liposomes attenuates experimental pulmonary fibrosis

**DOI:** 10.1186/s12967-022-03740-w

**Published:** 2022-11-12

**Authors:** Demin Cheng, Ziwei Li, Yue Wang, Haojie Xiong, Wenqing Sun, Siyun Zhou, Yi Liu, Chunhui Ni

**Affiliations:** 1 Department of Occupational Medical and Environmental Health, Key Laboratory of Modern Toxicology of Ministry of Education, School of Public Health, Nanjing Medical University, 818 Tianyuan East Road, 211166 Nanjing, China; 2 Gusu School, Nanjing Medical University, 818 Tianyuan East Road, 211166 Nanjing, China

**Keywords:** Liposomes, ZNF416, p-Smad2/3, Matrix stiffness, Pulmonary fibrosis

## Abstract

**Background:**

Pulmonary fibrosis is a chronic progressive fibrotic interstitial lung disease characterized by excessive extracellular matrix (ECM) deposition caused by activated fibroblasts. Increasing evidence shows that matrix stiffness is essential in promoting fibroblast activation and profibrotic changes. Here, we investigated the expression and function of matrix stiffness-regulated ZNF416 in pulmonary fibrotic lung fibroblasts.

**Methods:**

1 kappa (soft), 60 kappa (stiff) gel-coated coverslips, or transforming growth factor-beta 1 (TGF-β1)-cultured lung fibroblasts and the gain- or loss- of the ZNF416 function assays were performed in vitro. We also established two experimental pulmonary fibrosis mouse models by a single intratracheal instillation with 50 mg/kg silica or 6 mg/kg bleomycin (BLM). ZNF416 siRNA-loaded liposomes and TGF-β1 receptor inhibitor SB431542 were administrated in vivo.

**Results:**

Our study identified that ZNF416 could regulate fibroblast differentiation, proliferation, and contraction by promoting the nuclear accumulation of p-Smad2/3. Besides, ZNF416 siRNA-loaded liposome delivery by tail-vein could passively target the fibrotic area in the lung, and co-administration of ZNF416 siRNA-loaded liposomes and SB431542 significantly protects mice against silica or BLM-induced lung injury and fibrosis.

**Conclusion:**

In this study, our results indicate that mechanosensitive ZNF416 is a potential molecular target for the treatment of pulmonary fibrosis. Strategies aimed at silencing ZNF416 could be a promising approach to fight against pulmonary fibrosis.

**Supplementary Information:**

The online version contains supplementary material available at 10.1186/s12967-022-03740-w.

## Introduction

Pulmonary fibrosis is a progressive, lethal fibrotic lung disease with unknown causes characterized by sustained production and deposition of extracellular matrix (ECM). As the key effector cells, fibroblasts are responsible for the maintenance and remodeling of the ECM [1]. Persistent fibroblast differentiation into myofibroblast leads to impaired functional alveolar tissue architecture, which is indispensable for the progression of pulmonary fibrosis [2]. Certain evidence has shown that altered matrix stiffness mechanical properties are major divers of tissue fibrogenesis via promoting mechano-activation of fibroblasts [3].

Matrix stiffness is a central regulator of pulmonary fibrosis. The progression of organ fibrosis is often associated with increased tissue stiffness, mainly driven by activated fibroblasts depositing ECM proteins, including collagen, elastin, fibronectin, and hyaluronic acid [4]. Fibroblasts respond to mechanical cues of their extracellular surroundings both in vitro and in vivo, leading to fibroblast activation [5, 6]. Accumulating evidence suggests that external mechanical stimuli are translated into intracellular biochemical cascades that eventually focus on several transcription factors [7]. Yes-associated protein (YAP), the downstream effector of the Hippo signaling cascade, and Myocardin-related transcription factor A (MRTF-A) are known for mechanosensing transcriptional regulators regulated by matrix rigidity and implicated in fibroblast function [8, 9]. It is currently unknown whether other transcriptional regulators could target matrix stiffness associated with mechano-fibrogenic fibroblast activation for treating pulmonary fibrosis.

ZNF416 is a crucial histone acetyltransferase, which contains 12 tandem C2H2 zinc-finger domains and a KRAB domain, enabling it to act as either a transcriptional activator or repressor [10, 11]. Genome occupancy analysis using ChIP-seq suggested that ZNF416 responses to higher matrix stiffness induced fibroblast activation independent of other established fibrotic transcriptional regulators like YAP and serum response factor (SRF) [12]. However, the underlying molecular mechanism by which ZNF416 protects against pulmonary fibrosis in vivo and in vitro is still unclear and requires further exploration.

In this study, we observed that the expression of ZNF416 was upregulated in the fibrotic lesions of both human idiopathic pulmonary fibrosis (IPF) and silicosis. In vitro studies demonstrated that ZNF416 mediates p-Smad2/3 nuclear accumulation regulating fibroblast differentiation, proliferation and contraction. Furthermore, we utilized ZNF416 siRNA-loaded liposomes by the tail vein significantly to reverse the established pulmonary fibrosis induced by silica and bleomycin (BLM). Additionally, co-administration of ZNF416 siRNA-loaded liposomes and SB431542 further attenuated experimental pulmonary fibrosis. Here, our study may shed light on a new mechanosensing molecular mechanism and potential therapeutic target against pulmonary fibrosis.

## Materials and methods

### Animal models

Animal protocols were approved by the Ethical Committee of Nanjing Medical University. C57BL/6 mice (6–8 weeks old) were purchased from the Nanjing Medical University Animal Center (Nanjing, China). All animals were randomly assigned using Excel 2010 software random number formula. For silica or BLM instillation, mice were anesthetized and intratracheally injected with silica particles (50 mg/kg, Sigma-Aldrich, St. Louis, MO, USA) and BLM (6 mg/kg, MKBio Technology Co., Ltd., Shanghai, China). ZNF416 siRNA-loaded or scrambled liposomes (1 mg/kg, Ruixi Biological Technology Co., Ltd, Xi An, China) were injected into the anesthetized animals by tail vein on days 7, 14 and 21 following silica administration (on days 7 and 14 following BLM administration). For ZNF416 siRNA-loaded liposomes and the SB431542 co-administration group, mice have treated with ZNF416 siRNA-loaded liposomes once a week combined with 1 mg/kg of SB431542 (MedChemExpress) three times a week. Finally, the mice were euthanized on day 28 following the silica (on day 21 following BLM) treatment to analyze pulmonary fibrosis.

### Human lung samples

Human lung tissues were collected from patients with IPF, silicosis, and normal in Nanjing Drum Tower Hospital. Informed consent forms were signed by donors or their families before sample collection. The experiments were approved by the Human Assurance Committee of Nanjing Medical University (Nanjing, China).

### Histopathological analysis

Lung samples were fixed in 4% paraformaldehyde, embedded in paraffin and then sectioned (5 μm) to expose the main intrapulmonary bronchus. For histopathological analysis, the sections were then stained with hematoxylin and eosin (H&E), Masson’s trichrome stain and Sirius red to evaluate the degree of fibrosis.

### Immunofluorescence and immunohistochemistry staining

Formalin-fixed, cryo-embedded mouse lung sections were incubated overnight with α-SMA, Collagen I, ZNF416 and p-Smad2/3 primary antibody at 4℃followed by incubation with fluorophore-conjugated secondary antibodies for 1 h at room temperature. Nuclei were stained with DAPI. Immunofluorescence images were obtained using a fluorescence microscope (Olympus, Tokyo, Japan). For cellular immunofluorescence staining, cells were labeled with indicated antibodies overnight and then incubated with fluorophore-conjugated secondary antibodies. Nuclei were stained with DAPI. To visualize actin filaments, cells were incubated with Actin-Tracker Red-Rhodamine (Beyotime, China) for 30 min at room temperature.

Paraffin-embedded human lung tissue sections were subjected to immunostaining. After deparaffinization and antigen retrieval, the lung tissue sections were incubated overnight with ZNF416 antibodies, followed by scanning with Pannoramic Scanning Electron Microscope. The primary antibodies for immunofluorescence and immunohistochemistry analysis are specified in Additional file 1: Table S1.

### Hydroxyproline content assay

Lung collagen deposition was assessed by measuring the hydroxyproline content of lung homogenates with a hydroxyproline assay kit (A030-2, Jiancheng Bioengineering Institute, Nanjing, China) following the manufacturer’s protocol. The optical density of each sample and standard was measured at 550 nm, and the concentration of lung Hyp was calculated from a Hyp standard curve.

### Preparation and characterization of Znf416 siRNA-loaded liposomes

Liposomes were performed as carriers to encapsulate siRNA. A lipid solution in which the lipidoid, cholesterol, DSPC and mPEG-DMG at a molar ratio of 50:38.5:10:1.5 were dissolved in a solution of 90% ethanol and 10 mM sodium citrate was prepared. Then, siRNA was dissolved in 10 mM citrate buffer, and the lipid components were mixed with the dissolved siRNA by vortexing such that the final weight ratio of lipidoid:siRNA was 5:1. The next step was ultrafiltration centrifugation to exclude free siRNA. Finally, the siRNA-liposomes were diluted in PBS. The hydrodynamic diameter, polydispersity, zeta potential and stability of the liposomes were measured by dynamic light scattering (DLS) (Malvern Zetasizer Nano-ZS, UK). A RiboGreen assay was employed to calculate the siRNA entrapment efficiency. The characteristics of liposomes (hydrodynamic diameter, zeta potential, morphology and stability) were listed in Fig. 6. After staining with 2% phosphotungstic acid, the liposomes were characterized by transmission electron microscopy (TEM, Jeol, Japan). To detect the biodistribution of the liposomes, mice received a single tail vein injection with 1 mg/kg of DiR labelled liposomes. The mice were then anesthetized and imaged by an in vivo imaging system (IVIS Spectrum, PerkinElmer, USA).

**Fig. 1:**
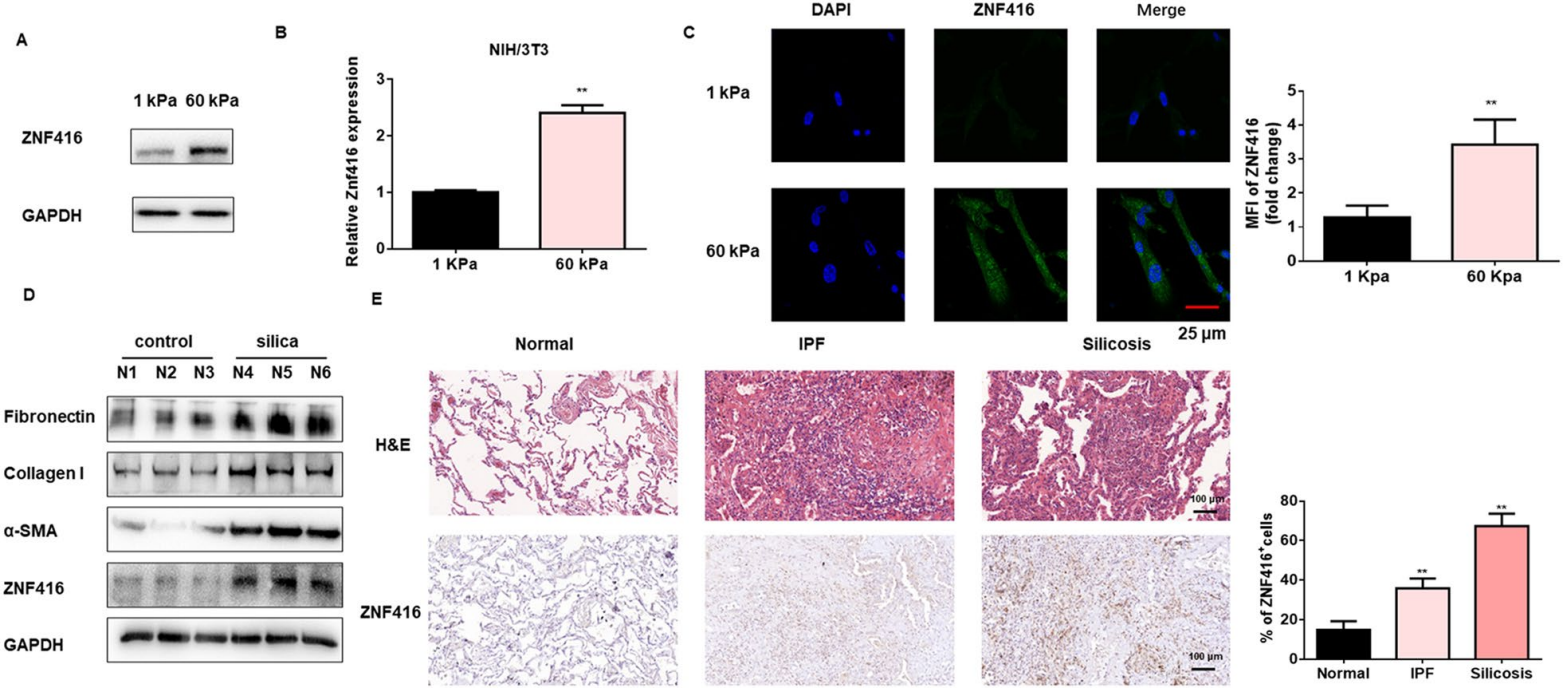
ZNF416 expression is increased in mechanics-induced fibroblasts and fibrotic lung tissues. NIH/3T3 cells were cultured on 1 or 60 kappa collagen-coated hydrogels coverslips. **A** Protein levels of ZNF416 were determined by western blot. **B** mRNA levels of ZNF416 were determined by qRT-PCR, with ^**^p < 0.01 vs. the 1 kappa group. **C** Immunofluorescence analysis and mean fluorescence intensity of ZNF416 in NIH/3T3 cells culture on 1 or 60 kappa collagen-coated hydrogels coverslips. ZNF416 stained green, Nuclei were stained by DAPI, scale bar = 25 μm, with ^**^p < 0.01 vs. 1 kappa group. **D** Western blot analysis of ZNF416 and fibrotic markers including Fibronectin, Collagen I and α-SMA expression in mouse lungs of control (n = 3) and silicosis (n = 3) group. **E** Representative images of H&E staining and ZNF416 expression, and semiquantitative scoring of ZNF416-positive cells, as obtained from immunohistochemistry images in normal, silicosis or IPF lung tissues of human subjects by IHC, scale bar = 100 μm, with ^**^p < 0.01 vs. the normal group

### Cell culture and treatment

The human fibroblast (MRC-5) and mouse fibroblast (NIH/3T3) were obtained from American Type Culture Collection (ATCC, Manassas, VA, USA). The MRC-5 cells were cultured in Minimum Essential Medium (MEM, Life Technologies/Gibco, Grand Island, NY, USA) supplemented with 10% FBS, penicillin/streptomycin and glutamine, while the NIH/3T3 cells were cultured in Dulbecco’s modified Eagle’s medium (DMEM) supplemented with 10% FBS, penicillin/streptomycin and glutamine. All of the cultures were incubated at 37  °C in 5% CO2. Cells were treated with 5 ng/ml recombinant TGF-β1 (Peprotech) for 48 h to induce fibroblast activation.

For 2D cell culture and treatments: the 2D Col-gel was acquired from Bioruo (Beijing, China). Following overnight serum deprivation (DMEM without FBS), NIH-3T3 cells were seeded on coverslips or plastic dishes coated with either 1 kappa (soft) or 60 kappa (stiff) polyacrylamide hydrogels with sterile collagen to create a uniform, thin layer of ECM protein according to the manufacturer’s protocol.

ZNF siRNA and control siRNA were synthesized by GenePharm (Shanghai, China). Lentiviral expression plasmids for ZNF416 were purchased from OriGene. Cells were transfected using riboFECTCP Reagent (Ribobio, Guangzhou, China) according to the manufacturer’s protocol. The sequences of ZNF416 siRNA were shown as follows: sense: CCUCAUACUUCUAGACCUCTT; antisense: GAGGUCUAGAAGUAUGAGGTT.

### Quantitative RT‑PCR (qRT‑PCR)

Total RNA was isolated from lung tissues and fibroblasts with TRlzol reagent (Takara, Dalian, China). qRT‑PCR assays were performed using HiScript II Q Select RT Supermix (Vazyme Biotech Co, Nanjing, China) and AceQ® qPCR SYBR® Green Master Mix kit (Vazyme Biotech Co., Ltd., Nanjing, China), according to the manufacturer’s instructions. The RNA quantity and quality were measured using a NanoDrop 2000 spectrophotometer (Thermo Scientific, MA, USA). The RNA levels of GAPDH were used to normalize the data. The primer sequences were provided in Additional file 1: Table S2.

### Western blot assay

Protein expressions were determined by western blotting. Proteins (100 µg) were separated by SDS-PAGE, electrophoretically transferred to nitrocellulose membranes and blocked for 1 h in PBS containing Tween 20 (0.1%) and non-fat milk (5%). The blots were incubated with the antibodies at 4   °C overnight. The membranes were then incubated for 1 h with horseradish peroxidase-conjugated secondary antibodies (1:1000, Beyotime, China). The specific details of the primary antibodies are shown in Additional file 1: Table S1. Using ChemiDocXRS + imaging system (Bio-Rad Laboratories, Inc) exposing blots and analyzing them.

### Cell proliferation and viability assay

Cells were cultured in 96-well plates at a density of 2 × 10^3^ cells/well. Using the EdU proliferation kit (Ribobio, Guangzhou, China) to detect cell proliferation, according to the manufacturer’s instructions. Briefly, 12 h after being seeded in the plates, cells were labeled with EdU for 2 h at 37  °C, treated with 100 µL of Apollo reaction cocktail and stained with 100 µL of Hoechst 33,342. Finally, the cells were observed under a fluorescence microscope (Olympus, Tokyo, Japan).

The cell proliferation curve was detected using the 3-[4,5-dimethylthiazol-2-ly]-2,5-diphenyltetrazolium bromide (MTT) kit (Beyotime, China). Briefly, NIH/3T3 cells were seeded in 96-well plates. The cells were transfected with ZNF416 siRNA for 2, 6, 12, 24, 36, 48-hour before the MTT assays. The optic density (OD) values were measured at 570 nm by a micro-plate reader, to analyze the proliferation rates of NIH/3T3 cells.

Cell viability was detected using the cell counting kit 8 (CCK8) assay, according to the manufacturers’ instructions. The absorbance was measured at 450 nm using a microplate reader (Bio-Tek EL, USA), and then the cell viability was calculated.

### Coimmunoprecipitation assay

Cells were transfected with the indicated treatment and then lysed with immunoprecipitation (IP) lysis buffer (RIPA lysis buffer and phenylmethylsulfonyl fluoride). After centrifugation, the supernatant lysates were subjected to IP assay with protein G-agarose beads (Beyotime, China). Then the beads were washed with cold PBS 3–5 times and boiled with 2 × SDS loading buffer at 4℃ before immunoblotting.

### Collagen gel contraction assay

NIH/3T3 cells were seeded in collagen gel (1 mg/mL, 200,110, Xinyou Biotechnology Co., Ltd) solution and incubated at room temperature for 20 min and the gel was allowed to solidify. The gel was then incubated in 1% FBS cell culture medium (depending on cell type) for five days and treated as indicated. Gels were imaged at the end of the experiment and analyzed using Image J software.

Serum biochemical determinations.

Blood obtained from mice was preserved at 4℃ overnight to allow the complete formation of the blood clot. The supernatants were then centrifuged at 3500 rpm for 5 min at 4℃ to obtain serum. Alanine aminotransferase/pyruvate transaminase (ALT/GPT) and aspartate aminotransferase (AST), the serum levels of creatinine (Scr), blood urea nitrogen (BUN) and creatine kinase-MB (CK-MB) (Jiancheng Bioengineering Institute, Nanjing, China) were measured in serum following manufacturer’s instructions.

### Statistical analysis

All statistical analyses were performed using GraphPad Prism (version 6.01, San Diego, CA, USA). Standard statistical analysis was applied to all the figures as appropriate and indicated in the figure legends. The data are presented as the mean ± SEM. In all cases, p < 0.05 was considered with statistical significance.

## Results

### ZNF416 expression is increased in mechanics induced fibroblasts and fibrotic lung tissues

To explore mechanotransduction in fibroblasts, we established a model of substrate mechanics in *vitro* by culturing NIH/3T3 fibroblasts on 60 kappa (stiff) or 1 kappa (soft) collagen-coated hydrogels coverslips. Cells cultured on the stiff matrix substrates increased ZNF416 expression at both the protein and mRNA levels (Fig. 1A, B). ZNF416 immunostaining showed high expression in cells cultured on 60 kappa gel-coated coverslips compared with those cultured on coverslips coated with 1 kappa (Fig. 1C). These results indicated that ZNF416 expression is increased in matrix stiffness-induced lung fibroblasts.

**Fig. 2:**
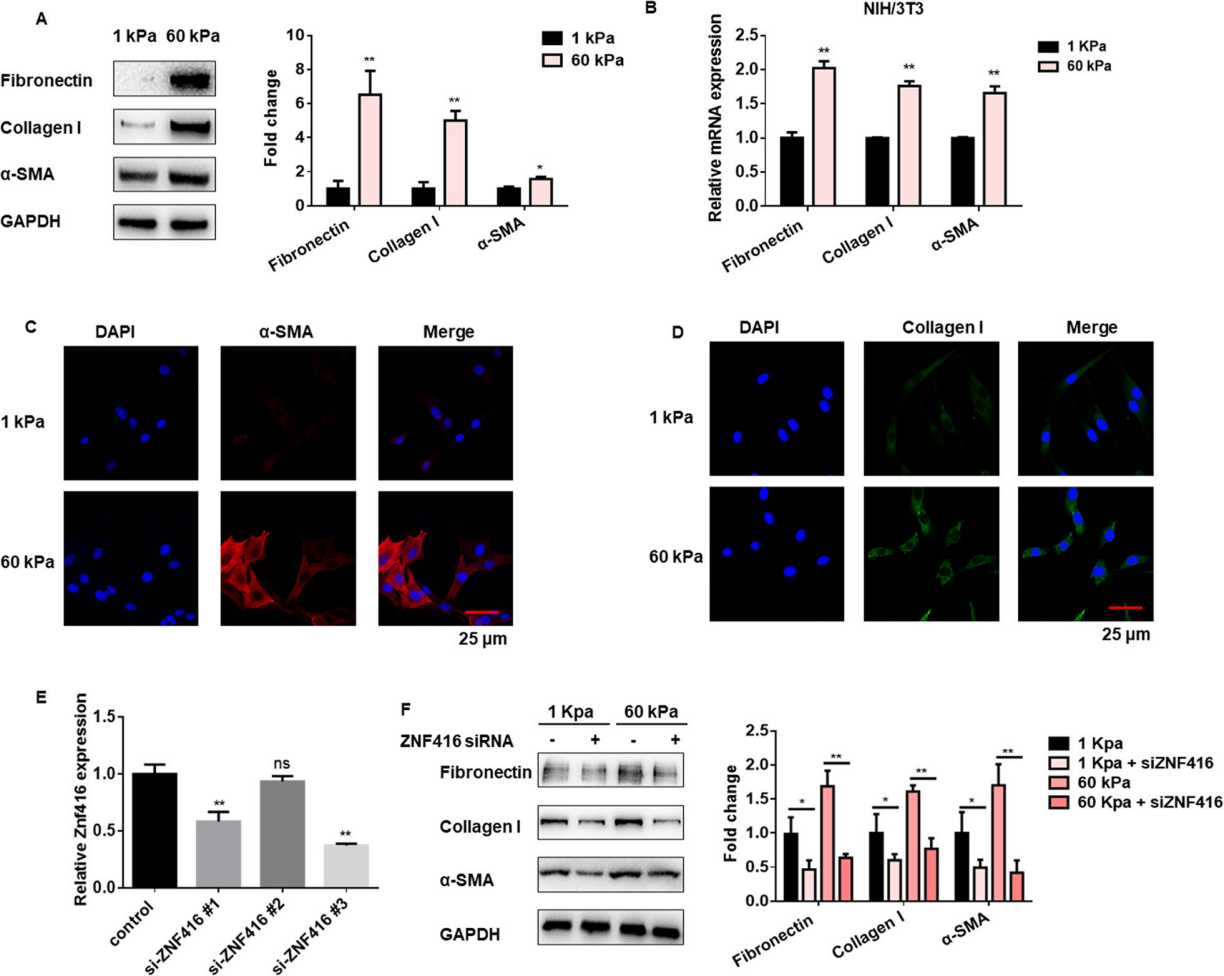
Increasing matrix stiffness induced fibroblast activation via ZNF416. NIH/3T3 cells were cultured on 1 or 60 kappa collagen-coated hydrogels coverslips. **A** Protein levels and densitometric analysis of fibrotic markers were determined by western blot, with ^**^p < 0.01 vs. the 1 kappa group. **B** mRNA levels of fibrotic markers were determined by qRT-PCR, with ^**^p < 0.01 vs. the 1 kappa group. **C**, **D** Immunofluorescence analysis of α-SMA and Collagen I in NIH/3T3 cells culture on 1 or 60 kappa collagen-coated hydrogels coverslips. α-SMA stained red, Collagen I stained green, Nuclei were stained by DAPI, scale bar = 25 μm. **E** qRT-PCR showed the silencing efficacy of siRNAs against ZNF416, with ^**^p < 0.01 vs. the control group. **F** NIH/3T3 cells culture on 1 or 60 kappa collagen-coated hydrogels coverslips in the presence of ZNF416 siRNA or the control siRNA. Levels of fibrotic markers were determined by western blot

We further investigated the expression of ZNF416 in fibrotic lung tissues. Increased ZNF416 expression was detected in the lung tissues of mice following silica and BLM injection than in those of untreated mice (Additional file 1: Fig. S1A). Consistent results were also obtained by western blot analysis of ZNF416 expression coupled with higher expression levels of Fibronectin, Collagen I, and α-SMA, which are markers of fibrosis (Fig. 1D and Additional file 1: Fig. S1B). Next, we examined the expression of ZNF416 in the lung sections of fibrotic patients and control subjects. Immunohistochemical (IHC) analysis indicated higher expression of ZNF416 in the lung tissues of both IFP and silicosis patients, whereas little ZNF416 expression was found in the normal subjects (Fig. 1E). Additionally, the immunostaining assay showed increased expression of α-SMA and ZNF416 in the fibrotic mouse lung tissues (Additional file 1: Fig. S1C, D). Taken together, these data suggested that ZNF416 expression is also increased in the fibrotic lung tissues.

### Increased matrix stiffness induced fibroblast activation via ZNF416

To test whether matrix stiffness regulates fibroblast activation, we detected the expression of fibrotic markers. NIH/3T3 cells seeded on 60 kappa hydrogels showed higher levels of fibrotic markers in both protein and mRNA levels compared with cells cultured on 1 kappa gels (Fig. 2A, B). Consistent with this finding, the stiff matrix enhanced the α-SMA and Collagen I staining as revealed by immunostaining assays (Fig. 2C, D and Additional file 1: Fig. S2A-B). Activated fibroblasts were responsive to the matrix stiffness, as shown by Actin staining (a primary mechanical cue) in the NIH/3T3 fibroblasts (Additional file 1: Fig. S2C). Next, we constructed three small interference RNAs (siRNAs) against ZNF416 to silence its expression in NIH/3T3 cells. qRT-PCR and western blot results showed that siRNA-3 could effectively inhibit the expression of ZNF416 cultured on 1 or 60 kappa hydrogels (Fig. 2E and Additional file 1: Fig. S2D). Then, we applied siRNA-3 to examine the role of ZNF416, and qRT-PCR results showed that silencing of ZNF416 inhibited the expression of Fibronectin, Collagen I, and α-SMA in NIH/3T3 cells cultured on 1 kappa hydrogels (Additional file 1: Fig. S2E). At the same time, the results from western blot showed that ZNF416 inhibition alleviated matrix stiffness-induced expression of fibrotic markers (Fig. 2F). Together, these data suggest that the stiff matrix promotes fibroblast activation via ZNF416.

**Fig. 3:**
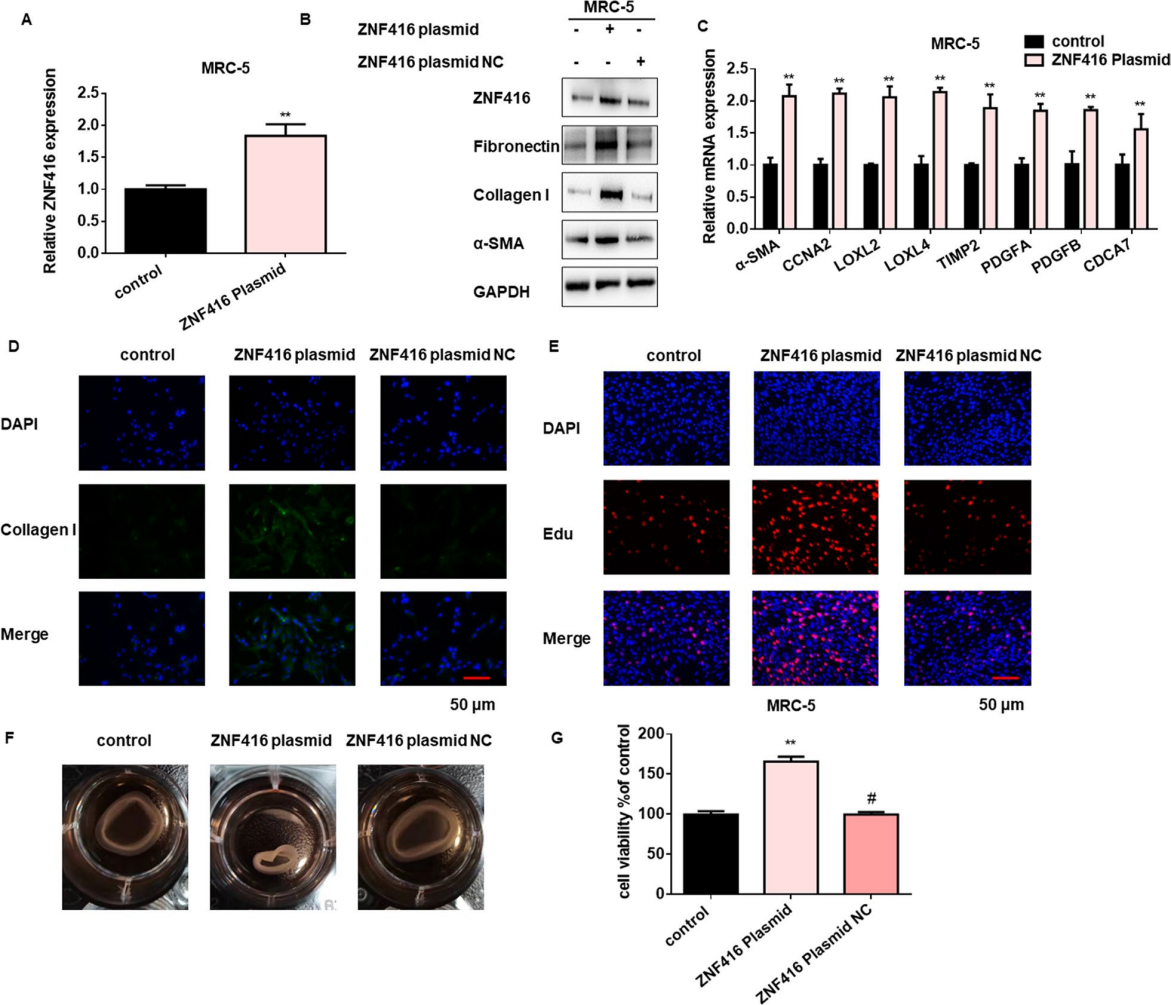
Gain- of function ZNF416 facilitates fibroblast differentiation, proliferation and contraction. **A** qRT-PCR analysis of relative expression of ZNF416 in MRC-5 cells treated with ZNF416 plasmid, with ^**^p < 0.01 vs. control group. **B** Western blots demonstrated a dramatically increased synthesis of fibrosis-related proteins after being treated with the ZNF416 plasmid in MRC-5 cells. **C** qRT-PCR demonstrated a dramatically increased expression of fibrosis-related genes after treatment of ZNF416 plasmid in MRC-5 cells. **D** Immunofluorescence analysis of Collagen I after treatment of ZNF416 plasmid in MRC-5 cells, Collagen stained green, Nuclei were stained by DAPI, scale bar = 50 μm. **E** EdU assays indicated that ZNF416 overexpression promotes the proliferation of MRC-5 cells, scale bar = 50 μm. **F** Effect of ZNF416 plasmid on the contractility of fibroblast. **G** CCK8 assays were performed to evaluate cell proliferative ability in MRC-5 cells after ZNF416 overexpression, with ^**^p < 0.01 vs. control group and ^#^*p* < 0.05 vs. ZNF416 plasmid group

### ZNF416 facilitates fibroblast differentiation, proliferation, and contraction

We further conducted gain- and loss-of-function studies to examine the potential role of ZNF416 in fibroblast function. We transfected the ZNF416 plasmid into fibroblasts to determine its potential profibrotic effect (Fig. 3A and Additional file 1: Fig. S3A). As expected, western blot results showed that ZNF416 overexpression could significantly promote the protein expression of fibrotic markers in fibroblasts (Fig. 3B and Additional file 1: Fig. S3B-C), which also amplified mRNA expression of some fibrotic target genes (Fig. 3C). By using immunofluorescence experiments, we found that ZNF416 overexpression could significantly increase the expression of Fibronectin and Collagen I (Fig. 3D and Additional file 1: Fig. S3D–F). In addition, overexpression of ZNF416 could dramatically promote fibroblast proliferation (Fig. 3E and Additional file 1: Fig. S3G). Fibroblast contraction assay showed that treatment with ZNF416 plasmid increased the collagen contractility capacity of fibroblast compared with the control group (Fig. 3F). Additionally, we performed cck8 assays and confirmed that ZNF416 overexpression increased cell viability (Fig. 3G).

**Fig. 4:**
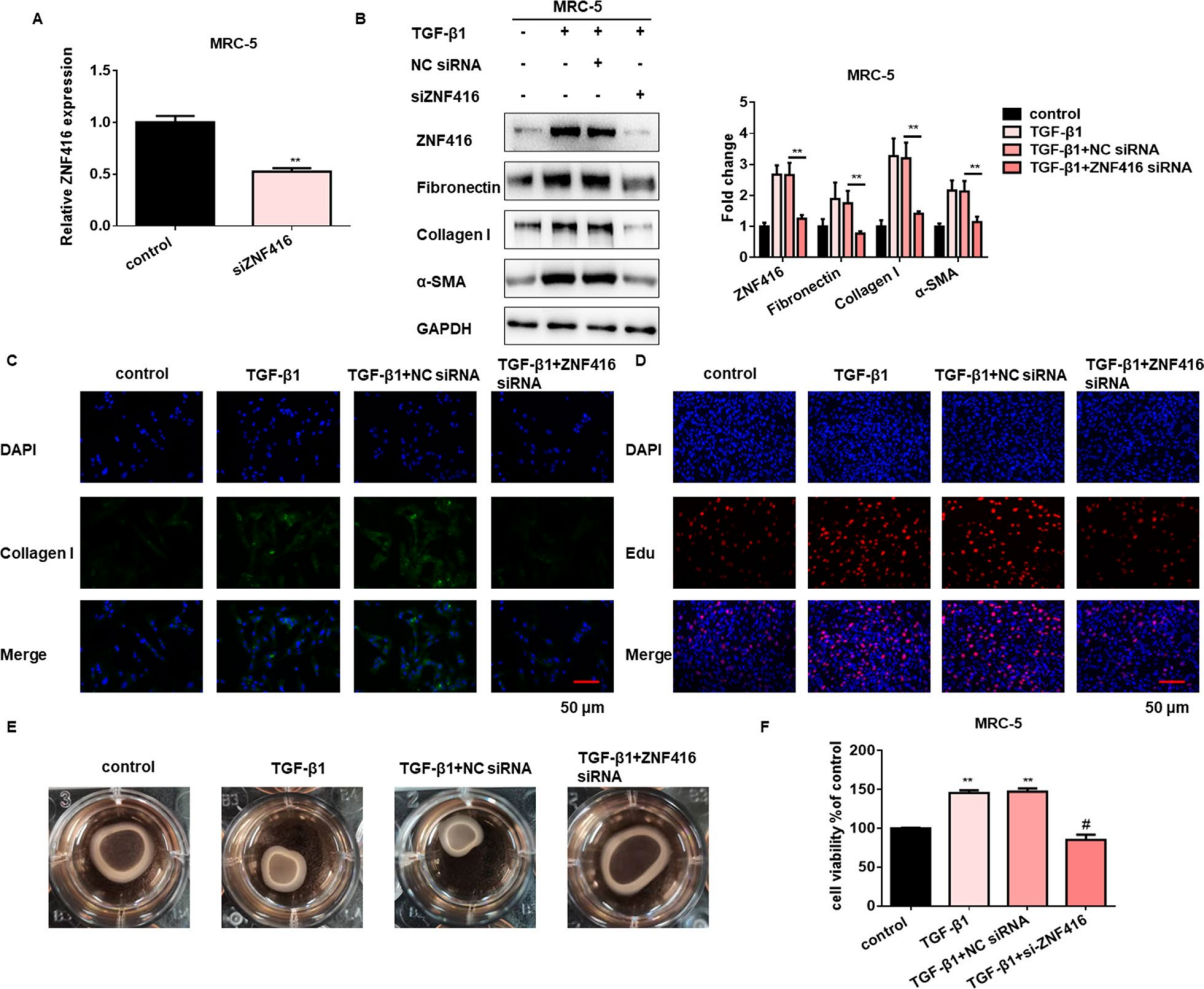
Loss- of function ZNF416 attenuates fibroblast differentiation, proliferation and contraction. **A** qRT-PCR analysis of relative expression of ZNF416 in MRC-5 cells treated with ZNF416 siRNA, with ^**^p < 0.01 vs. control group. **B** Western blots and densitometric analysis demonstrated a dramatically decreased expression of fibrosis-related proteins after treatment of ZNF416 plasmid in MRC-5 cells, with ^**^p < 0.01 vs. TGF-β1 + NC siRNA group. **C** Immunofluorescence analysis of Collagen I after treated with ZNF416 siRNA in MRC-5 cells, Collagen stained green, Nuclei were stained by DAPI, scale bar = 50 μm. **D** EdU assays indicated that ZNF416 siRNA attenuates the proliferation of MRC-5 cells, scale bar = 50 μm. **E** Effect of ZNF416 siRNA on the contractility of TGF-β1 induced fibroblast. **F** CCK8 assays were performed to evaluate cell proliferative ability in MRC-5 cells after ZNF416 knockdown, with ^**^p < 0.01 vs. control group and ^#^*p* < 0.05 vs. TGF-β1 + NC siRNA group

To further explore the loss-of-function of ZNF416 on fibroblasts, we evaluated the functions of ZNF416 after ZNF416 knockdown in the fibroblasts (Fig. 4A and Additional file 1: Fig. S4C). The results of the MTT assay showed that the relative proliferation of fibroblasts was gradually decreased in a time-dependent manner after ZNF416 knockdown (Additional file 1: Fig. S4A). Also, the cell viability of fibroblasts was suppressed under the treatment of ZNF416 siRNA alone (Additional file 1: Fig. S4B). Both western blot and qRT-PCR analyses demonstrated that fibroblast activation was abrogated in ZNF416 siRNA-transfected cells after TGF-β1 treatment (Fig. 4B and Additional file 1: Fig. S4D, E). Furthermore, immunofluorescence assays showed that inhibition of ZNF416 could significantly reduce the expression of Collagen I as well as Fibronectin induced by TGF-β1 (Fig. 4C and Additional file 1: Fig. S4F–H). Moreover, EdU proliferation and contraction assays found that silencing of ZNF416 expression remarkably reduced fibroblast proliferation and contractility capacity (Fig. 4D, E and Additional file 1: Fig. S4I). Also, we examined the viability of fibroblast and found that the loss of ZNF416 suppressed cell viability stimulated by TGF-β1 (Fig. 4F). Collectively, these data support the notion that ZNF416 expression is critical to fibroblast differentiation, proliferation, and contraction.

**Fig. 5:**
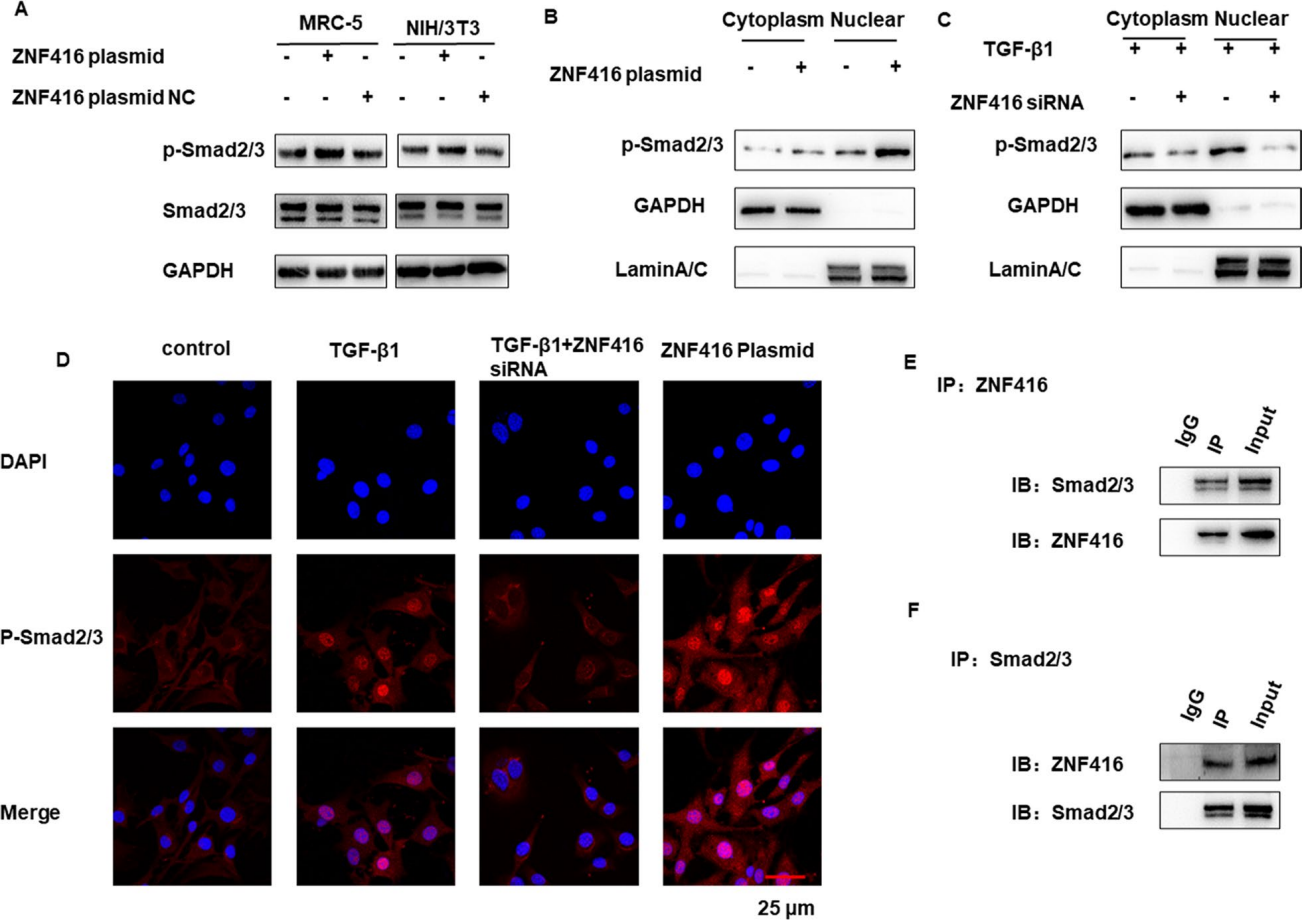
ZNF416 regulates the activation of fibroblast by promoting the nuclear accumulation of p-Smad2/3. **A** Protein levels of Smad2/3 and p-Smad2/3 were determined by western blot after ZNF416 overexpression. **B**, **C** Western blot analysis of the levels of p-Smad2/3 in the cytoplasm and nuclear after different treatments. **D** Subcellular localization of p-Smad2/3 in the indicated groups by immunofluorescence assays, p-Smad2/3 stained red, Nuclei were stained by DAPI, scale bar = 25 μm. **E**, **F** Coimmunoprecipitation of ZNF416 and Smad2/3 in NIH/3T3 cells

### ZNF416 regulates the activation of fibroblasts by promoting the nuclear accumulation of p-Smad2/3

TGF-β/Smad signaling pathway has been widely reported to involve in the activation of fibroblast activation in fibrogenesis [13]. Therefore, we examined the effects of ZNF416 on TGF-β1-induced Smad signaling. As a transcriptional regulator, we firstly detected the impact of ZNF416 on Smad2/3. However, the Smad2 and Smad2 mRNA levels were almost unchanged in the cells under ZNF416 different treatment (Additional file 1: Fig. S5A). Next, the impact of ZNF416 on the phosphorylation of Smad2/3 was detected. Impressively, treatment with the ZNF416 plasmid increased the phosphorylation of Smad2/3. However, the suppression of ZNF416 blocked the phosphorylation of Smad2/3 induced by TGF-β1 (Fig. 5A and Additional file 1: Fig. S5B). Since Smad2/3 translocation into the nucleus is critical for its function, we next estimated the localization of p-Smad2/3 in fibroblasts. As shown in Fig. 5B–D and Additional file 1: Fig. S5D, overexpression of ZNF416 promoted phosphorylated Smad2/3 nuclear translocation, while the suppression of TGF-β-mediated Smad2/3 phosphorylation nuclear translocation by ZNF416 knockdown. Furthermore, the co-immunostaining results showed that ZNF416 (green) was colocalized with p-Smad2/3 (red) in TGF-β1 treated fibroblasts (Additional file 1: Fig. S5C). Then, endogenous Co-IP assay in NIH/3T3 cells confirmed the interaction between ZNF416 and Smad2/3 (Fig. 5E, F). The above results suggested that ZNF416 mediates fibroblast activation by promoting the nuclear accumulation of p-Smad2/3.

**Fig. 6:**
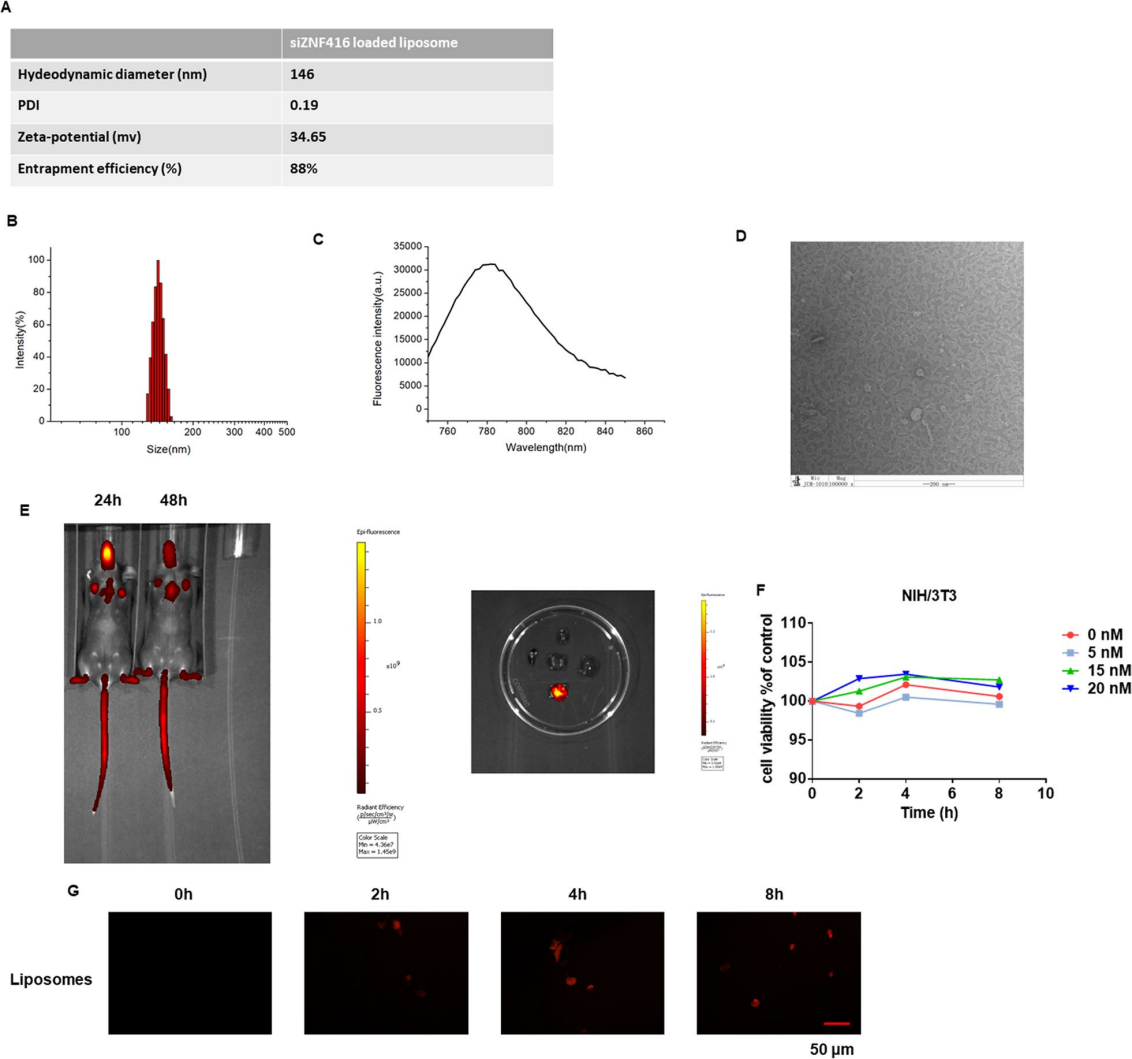
Characteristics of the ZNF416 siRNA- loaded liposomes. **A** Hydrodynamic diameter, PDI, zeta potential and entrapment efficiency of ZNF416 siRNA- loaded liposomes. **B**, **C** Size and fluorescence intensity of siRNA- loaded liposomes. **D** Representative TEM image of siRNA-loaded liposomes. **E** Representative IVIS images of the mouse administrated with DiR-labeled liposomes by tail vein and ex vivo fluorescence images of major organs from mice. **F** CCK8 assays were performed to evaluate the effect of siRNA-loaded liposomes on cells. **G** Immunofluorescence of siRNA-loaded liposomes in cells, DiR-labeled liposomes marked red

### Administration of ZNF416 siRNA-loaded liposomes attenuates pulmonary fibrosis in experimental mouse models

**Fig. 7:**
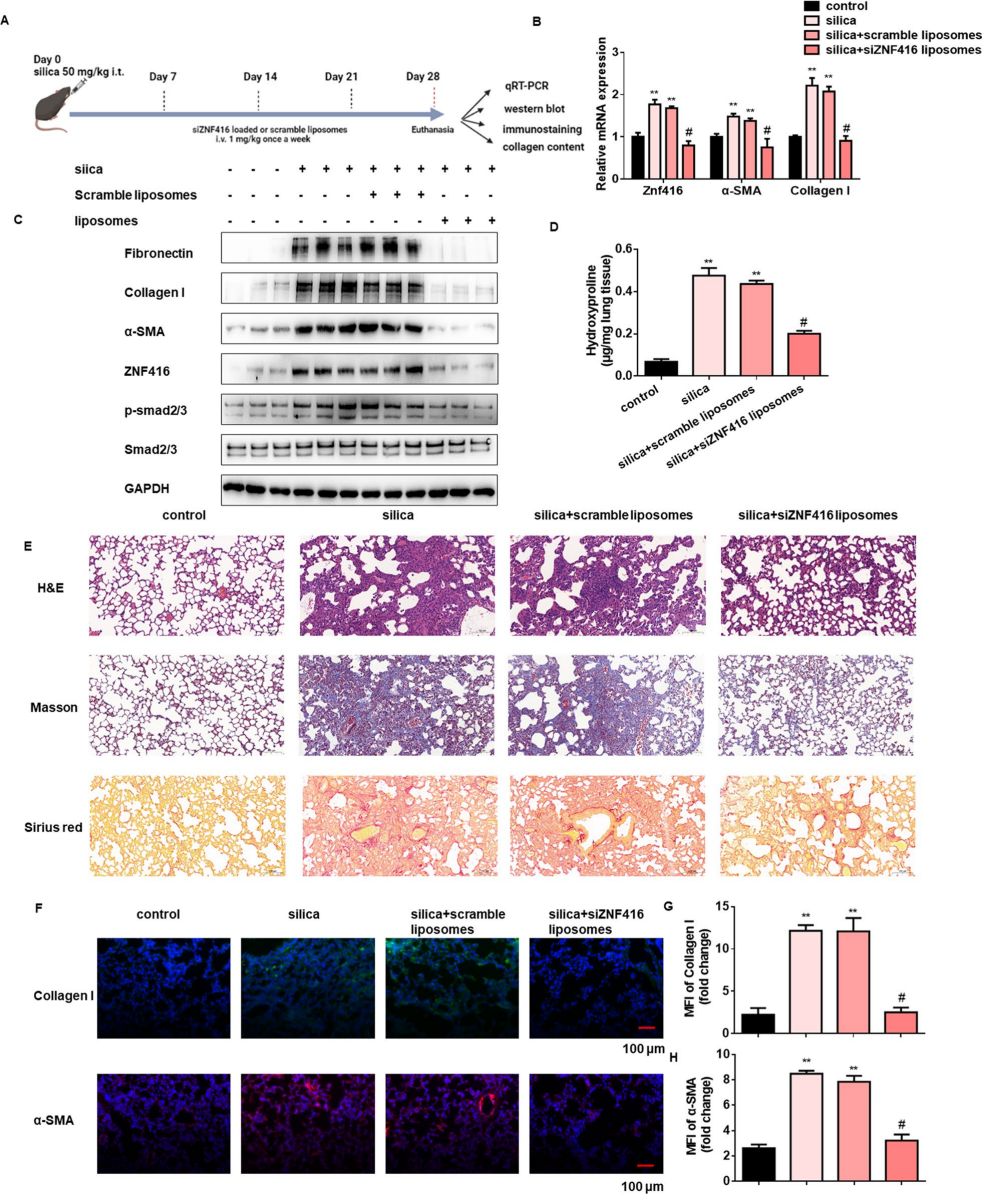
Administration of ZNF416 siRNA-loaded liposomes attenuates silica-induced mouse fibrosis. **A** Experimental design. 8-week-old C57BL/6 mice were injected intratracheally with silica (50 mg/kg) or saline. ZNF416 siRNA-loaded or scrambled liposomes (1 mg/kg) were injected into the anesthetized animals by tail vein on days 7, 14 and 21 following silica administration. **B** qRT-PCR analysis of relative expression of ZNF416, α-SMA and Collagen I in the indicated groups, with ^**^p < 0.01 vs. control group and and ^#^*p* < 0.05 vs. the scramble liposomes group. **C** Protein levels of fibrotic markers, ZNF416, Smad2/3 and p-Smad2/3 in the different groups. **D** Hydroxyproline levels in lungs of C57/BL6 mice from the different groups, with ^**^p < 0.01 vs. control group and ^#^*p* < 0.05 vs. the scramble liposomes group. **E** Representative hematoxylin and eosin (H&E) and Masson’s trichrome staining and Sirius red of lung sections from the indicated groups. scale bar = 100 μm. **F** Immunofluorescence staining with Collagen I and α-SMA in mouse lung slices from the different groups. scale bar = 100 μm. **G**, **H** Mean fluorescence intensity of Collagen I and α-SMA in lung slices from the different groups, with ^**^p < 0.01 vs. control group and ^#^*p* < 0.05 vs. the scramble liposomes group

To explore whether targeting ZNF416 could prevent further progression of established fibrosis in vivo, we used ZNF416 siRNA-loaded liposomes to treat silica- and BLM-induced mouse pulmonary fibrosis models ( Fig. 7A and Additional file 1: Fig. S7A). Recent studies have indicated that liposomes are widely used as drug carriers owing to their safety and ability to transport agents to target sites [14]. The prepared liposomes showed a 88% entrapment efficiency for loading ZNF416 siRNA with a zeta potential of 34.65 mV and hydrodynamic diameter of 146 nm (Fig. 6A). Besides, the liposome size distribution and fluorescence intensity were shown in Fig. 6B, C. A representative image was then taken by transmission electron microscope (TEM) (Fig. 6D). DiR-labeled liposomes were administrated to mice by tail vein to observe the biodistribution in the lung. Then fluorescence signal was imaged by IVIS and predominantly accumulated in the lung (Fig. 6E). We also assessed the effect of liposomes on fibroblasts and found that the liposomes could enter the fibroblasts without cytotoxicity (Fig. 6F, G). Besides, we measured a series of serum parameters, including alanine aminotransferase/pyruvate transaminase (ALT/GPT) and aspartate aminotransferase (AST), the serum levels of creatinine (Scr), blood urea nitrogen (BUN) and creatine kinase-MB (CK-MB) in control and ZNF416 siRNA-loaded liposomes treated mouse serum. We also found no significant differences in these markers of the liver, kidney, and heart function and body weight in comparison with controls (Additional file 1: Fig. S6A–F), and no major organ histopathological changes in H&E staining were observed either (Additional file 1: Fig. S6G).

Following this, we used ZNF416 siRNA-loaded liposomes in fibrotic lung mice to investigate the function of ZNF416 in silica-induced pulmonary fibrosis in vivo ( Fig. 7A). As illustrated in Fig. 7B C, the mRNA and protein levels of ZNF416, Col1age I, and α-SMA after silica treatment were significantly lower in the ZNF416 siRNA-loaded liposomes administration mice than in scramble-loaded liposomes. Notably, Hematoxylin and eosin (H&E), Masson trichrome and Sirius red staining of the lung sections from mice in the ZNF416 siRNA-loaded liposomes administration group demonstrated a remarkable decrease in lung parenchymal fibrotic lesions compared with scramble-loaded liposomes administration group, as evidenced by decreased levels of hydroxyproline (Fig. 7D and E). Furthermore, Collagen I and α-SMA both showed increased expression levels in the lung of silica-induced mice while reduced expression levels in silica-induced ZNF416 siRNA-loaded liposomes administration mice, as detected by the immunofluorescence staining (Fig. 7F–H). ​Consistently, the administration of ZNF416 siRNA-loaded liposomes could also attenuate BLM-induced pulmonary fibrosis, which was evidenced by qRT-PCR, western blot, H&E, Masson trichrome and Sirius red staining, immunofluorescence and hydroxyproline assays (Additional file 1: Fig. S7A–H). Collectively, the above results provide evidence of the in vivo ZNF416 siRNA-loaded liposomes administration in reversing experimental mouse pulmonary fibrosis models.

### Synergy therapeutic effect of ZNF416 siRNA-loaded liposomes and TGF-β1 receptor inhibitor on experimental pulmonary fibrosis in mice

**Fig. 8:**
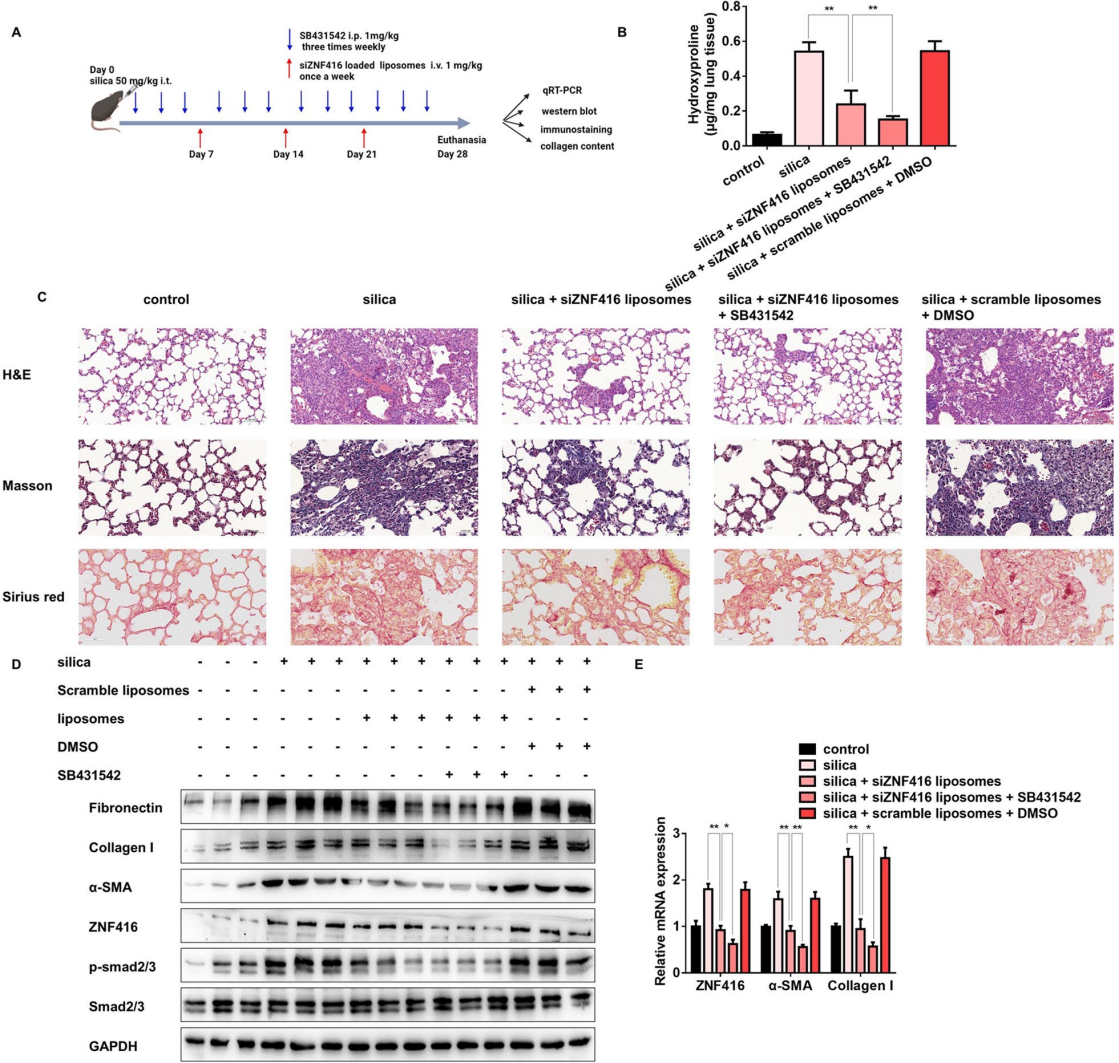
Combination of ZNF416 siRNA-loaded liposomes and SB431542 exerts a synergic effect on silica-induced pulmonary fibrosis. **A** Strategy for co-administration of ZNF416 siRNA-loaded liposomes and SB431542 in the silica-induced pulmonary fibrosis mouse model. **B** Quantification of hydroxyproline contents in mice after silica challenge for the indicated group, with ^**^p < 0.01 vs. the indicated group. **C** H&E staining, Masson staining, and Sirius red staining were performed to measure the severity of lung fibrosis. **D**,** E** Western blot and qRT‐PCR analysis of fibrotic markers and ZNF416 protein and mRNA level in mouse lung tissues on saline, SiO_2_, SiO_2_ + siZNF416 liposomes, SiO_2_ + siZNF416 liposomes + SB431542 and SiO_2_ + scramble liposomes + DMSO group, with ^**^p < 0.01 vs. the indicated group

To detect the synergistic effects of ZNF416 siRNA-loaded liposomes and TGF-β signaling in vivo, we made another intervention model by administration of ZNF416 siRNA-loaded liposomes and TGF-β1 receptor inhibitor SB431542 in silica and BLM-induced pulmonary fibrosis (Fig. 8A and Additional file 1: Fig. S8A). As expected, co-treatment of ZNF416 siRNA-loaded liposomes and SB431542 displayed much lower levels of hydroxyproline in the silica-induced lung tissues (Fig. 8B). Moreover, compared with the single treatment, the combination group showed exceedingly attenuated pulmonary injury and collagen deposition as illustrated by the histopathological analysis (Fig. 8C). Of note, ZNF416 siRNA-loaded liposomes combined with SB431542 effectively decreased the protein and mRNA levels of pro-fibrotic mediators (Fig. 8D, E). Besides the synergy therapeutic effect in silica-induced pulmonary fibrosis, treatment with BLM instillation further demonstrated the potential synergy effect of ZNF416 siRNA-loaded liposomes and SB431542 (Additional file 1: Fig. S8B–E). These results indicated that the combination of ZNF416 siRNA-loaded liposomes and SB431542 exerts a synergic effect on experimental pulmonary fibrosis.

## Discussion

**Fig. 9:**
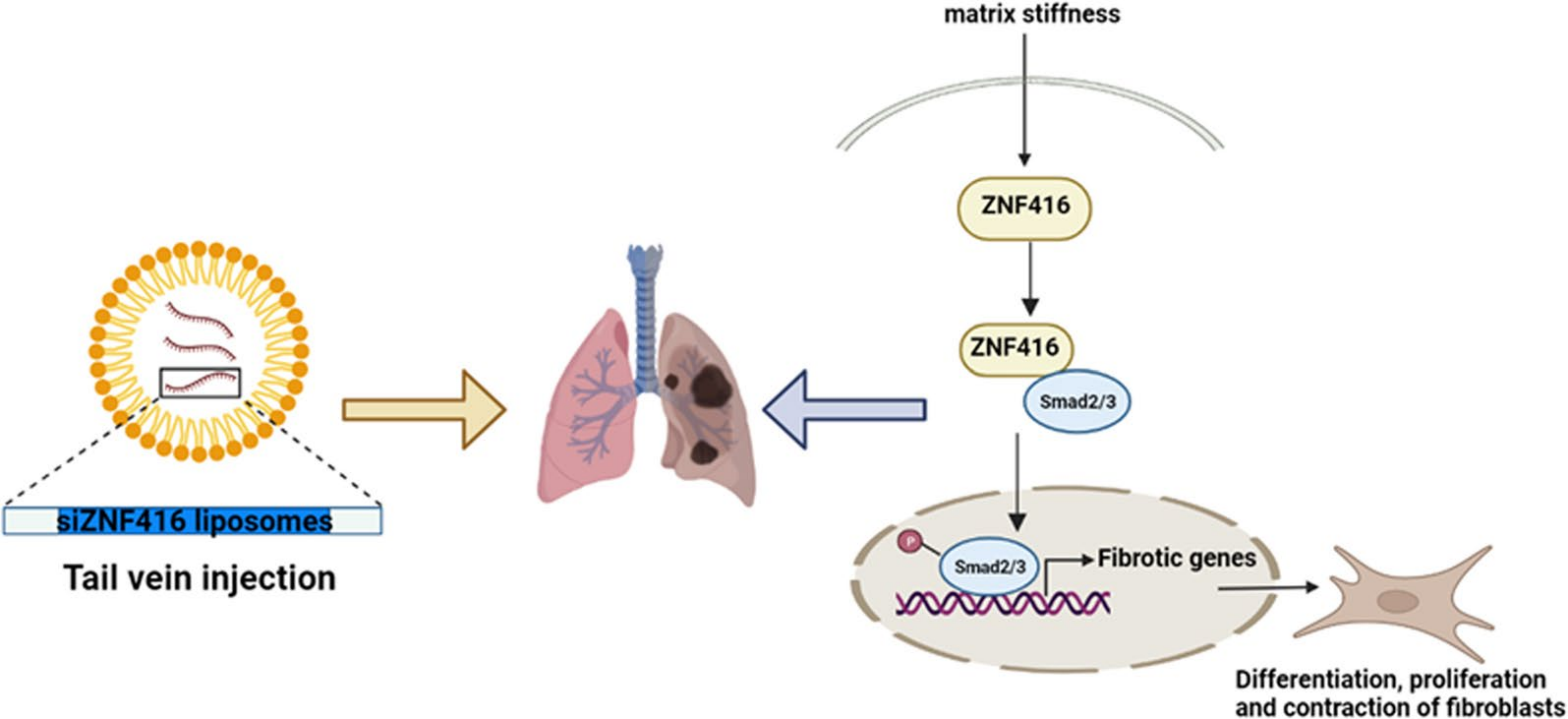
Schematic illustration of the mechanisms of ZNF416 in fibroblasts. Created with https://BioRender.com. ZNF416 is upregulated by matrix stiffness, after which ZNF416 is bound to Smad2/3 and promotes the nuclear accumulation of p-Smad2/3 to promote the differentiation, proliferation, and contraction of fibroblast. Besides, the treatment of mice with ZNF416 siRNA-loaded liposomes in *vivo* reverses established pulmonary fibrosis

Pulmonary fibrosis is an advanced interstitial lung disease characterized by excessive ECM deposition with poor prognosis [15, 16], including silicosis and IPF. Despite the current approved two antifibrotic medicine (nintedanib and pirfenidone) having been shown to reduce lung function decline, there is not enough to postpone fibrosis [17]. Recent results indicate that ZNF416 is a novel transcriptional regulator of fibroblast mechanoactivation [12], suggesting that this gene might be involved in matrix stiffness-induced fibrosis. In the present study, we investigated the function of ZNF416 as a matrix stiffness-regulated mechanosensitive regulator during pulmonary fibrosis. High levels of ZNF416 were detected in the fibrotic lung sections of human IPF and silicosis, as well as matrix stiffness-stimulated fibroblasts. Mechanistically, in vitro studies showed that increasing matrix stiffness upregulates ZNF416 expression. After which, ZNF416 bounds to Smad2/3 and promotes the nuclear accumulation of p-Smad2/3, promoting the differentiation, proliferation, and contraction of fibroblast (Fig. 9). The overexpression or knockdown of ZNF416 within fibroblasts aggravated or attenuated the profibrogenic phenotype in vitro, respectively. Most importantly, our results showed that using ZNF416 siRNA-loaded liposomes could alleviate pulmonary fibrosis in mouse fibrotic models, providing a promising antifibrotic target.

To date, matrix stiffness has been recognized as a pivotal regulator of tissue fibrogenesis that blocks the normal physiologic wound healing responses to promote organ fibrosis, such as matrix stiffness promotes the MDM4-p53 pathway resulting in pulmonary fibrosis[18, 19]. Besides, it has been reported that transcriptional and epigenetic regulators like Rho-associated coiled-coil forming kinase (ROCK) and nuclear localization of transcriptional regulators YAP mediating matrix stiffness induced organ fibrosis [20, 21]. Pulmonary fibrosis progression features ECM stiffening due to intra- and intermolecular cross-links and deposition of collagen, which dramatically induce abnormal ECM accumulation [22, 23]. Meanwhile, the mechanical properties of the ECM are resistant to proteolytic degradation or other ECM-degrading proteases, further increasing the matrix stiffness. Accumulating evidence indicates that the crosslink of stiffened ECM and fibrotic lung fibroblasts provide a feedforward mechanism that amplifies pulmonary fibrosis [24–26]. Understanding the key mechanosensitive molecules that contribute fibroblasts to myofibroblasts may uncover a promising strategy for the treatment of persistent and progressive pulmonary fibrosis.

ZNF416 contains diverse domains that confer dual properties, including a transcriptional activator or repressor. A recent study indicates that ZNF416 largely co-occupies sequences associated with H3K27ac, suggesting ZNF416 may act as a transcriptional activator [12]. In the present study, we reported that ZNF416 promoted the differentiation, proliferation and contraction of fibroblast. It is well known that phosphorylation of Smad2/3 translocates into the nucleus could promote fibrosis-associated gene expression and myofibroblast phenotype [27]. Therefore, we examined the effects of ZNF416 on Smad2/3 signaling in fibroblasts. It was likely that the effect of ZNF416 on Smad2/3 was not altered at the transcriptional level, as the overexpression or knockdown of ZNF416 did not change the mRNA level of Smad2 and Smad3. However, ZNF416 plasmid and ZNF siRNA robustly increased or decreased the protein level of p-Smad2/3. Mechanistically, ZNF416 regulates fibroblast activation by promoting the nuclear accumulation of p-Smad2/3. Further, the co-IP assay shows that ZNF416 could interact with Smad2/3 in fibroblast. We next investigated the expression of ZNF416 in fibrotic lung tissues. Interestingly, we illustrated that ZNF416 was increased in silicosis samples and experimental mouse pulmonary fibrosis models. Additionally, we next addressed whether targeting ZNF416 could be an attractive strategy for attenuating pulmonary fibrosis.

RNA interference (RNAi)-based therapy has emerged as a promising gene therapeutic strategy owing to its safety and ability to silence gene expression in a specific manner [28, 29]. Small double-stranded RNA molecules can deliver RNAi to trigger the silence of specific genes in cells or tissues with fewer side effects [30]. Currently, siRNA therapies used in clinical practice have faced the main challenge of lacking effective vectors which protect the siRNA against nuclease degradation [31]. Previous studies have established liposomes as RNAi carriers for their safety and higher distribution in the injured area [32, 33]. Herein, we showed that ZNF416 siRNA-loaded liposomes passively accumulated in the pulmonary fibrotic areas of mice following tail vein injection. Notably, administration of ZNF416 siRNA-loaded liposomes remarkably reversed established experimental mouse pulmonary fibrosis. Notably, administration of ZNF416 siRNA-loaded liposomes combined with TGF-β1 receptor inhibitor remarkably reversed experimental mouse pulmonary fibrosis.

## Conclusion

Our data suggest that ZNF416 is essential for fibroblast activation and pulmonary fibrosis progression. Therefore, ZNF416 siRNA-loaded liposomes could be a viable therapeutic approach for pulmonary fibrosis.

## Supplementary Information

**Additional file 1: Fig. S1**. ZNF416 expression is increased in mechanics-induced fibroblasts and fibrotic lung tissues. (A) mRNA levels of ZNF416 were determined by qRT-PCR in the mouse lung tissues of the control, silicosis and BLM group, with **p < 0.01 and *p < 0.05 vs. the control group. (B) Western blot and densitometric analysis of ZNF416 and fibrotic markers including Fibronectin, Collagen I and α-SMA expression in mouse lungs of the control (n = 3) and BLM (n = 3) group, with **p < 0.01 vs. the control group. (C-D) Immunofluorescence analysis of α-SMA and ZNF416 in mouse lung slices from the different groups. scale bar = 100 μm.** Fig. S2**. Increasing matrix stiffness induced fibroblast activation via ZNF416. (A-B) Mean fluorescence intensity of α-SMA and Collagen I in NIH/3T3 cells culture on 1 or 60 kappa collagen-coated hydrogels coverslips, with **p < 0.01 vs. 1 kappa group. (C) Immunofluorescence analysis of Actin in NIH/3T3 cells culture on 1 or 60 kappa collagen-coated hydrogels coverslips. Actin stained red, Nuclei were stained by DAPI, scale bar = 25 μm. (D) Western blot showed the silencing efficacy of siRNAs against ZNF416 in NIH/3T3 cells culture on 1 or 60 kappa collagen-coated hydrogels coverslips plus with ZNF416 #1-#3 siRNA. (E) qRT-PCR analysis of relative expression of fibrotic markers after ZNF416 knockdown culture on 1 kappa collagen-coated hydrogels coverslips, with **p < 0.01 vs. the 1 kappa group.** Fig. S3**. Gain- of function ZNF416 facilitates fibroblast differentiation, proliferation and contraction. (A) qRT-PCR analysis of relative expression of ZNF416 in NIH/3T3 cells treated with ZNF416 plasmid, with **p < 0.01 vs. control group. (B) The densitometric analysis demonstrated a dramatically increased synthesis of fibrosis-related proteins after treatment of ZNF416 plasmid in MRC-5 cells, with **p < 0.01 vs. control group and #p < 0.05 vs. ZNF416 plasmid group. (C) Western blot demonstrated a dramatically increased expression of fibrosis-related protein after treatment of ZNF416 plasmid in NIH/3T3 cells. (D-E) Immunofluorescence analysis of Fibronectin and Collagen I after treatment of ZNF416 plasmid in NIH/3T3 cells, Fibronectin stained red, Collagen stained green, Nuclei were stained by DAPI, scale bar = 50 μm. (F) Mean fluorescence intensity of Collagen I in MRC-5 cells treated with ZNF416 plasmid or ZNF416 plasmid NC, with **p < 0.01 vs. the control group and #p < 0.05 vs. the ZNF416 plasmid group. (G) Cell proliferation of MRC-5 cells transfected with ZNF416 plasmid or ZNF416 plasmid NC, with **p < 0.01 vs. the control group and #p < 0.05 vs. the ZNF416 plasmid group.** Fig. S4**. Loss- of function ZNF416 attenuates fibroblast differentiation, proliferation and contraction. (A) MTT assays were performed to evaluate cell proliferative ability in fibroblasts. (B) CCK8 assays were performed to evaluate the effect of ZNF416 siRNA on cells, with **p < 0.01 vs. the control group. (C) qRT-PCR analysis of relative expression of ZNF416 in NIH/3T3 cells treated with ZNF416 siRNA, with **p < 0.01 vs. control group. (D) Western blots analysis demonstrated a dramatically decreased expression of fibrosis-related proteins after treatment of ZNF416 siRNA in NIH/3T3 cells. (E) qRT-PCR demonstrated a dramatically decreased expression of fibrosis-related genes after treatment of ZNF416 siRNA in NIH/3T3 cells, with **p < 0.01 vs. control group. (F-G) Immunofluorescence analysis of Fibronectin and Collagen I after being treated with ZNF416 siRNA in NIH/3T3 cells, Fibronectin stained red, Collagen stained green, Nuclei were stained by DAPI, scale bar = 50 μm. (H) Mean fluorescence intensity of Collagen I in MRC-5 cells treated with different treatments, with **p < 0.01 vs. the control group and #p < 0.05 vs. the TGF-β1 group. (I) Cell proliferation of MRC-5 cells transfected with ZNF416 siRNA, with **p < 0.01 vs. the control group and #p < 0.05 vs. the the TGF-β1 group.** Fig. S5**. ZNF416 regulates the activation of fibroblasts by promoting the nuclear accumulation of p-Smad2/3. (A) mRNA levels of Smad2 and Smad3 were determined by qRT-PCR in NIH/3T3 cells treated with ZNF416 plasmid or ZNF416 siRNA. (B) Protein levels of Smad2/3 and p-Smad2/3 were determined by western blot after ZNF416 knockdown. (C) Immunofluorescence analysis of p-Smad2/3 after treatment with different treatments in MRC-5 cells, p-Smad2/3 stained red, ZNF416 stained green, Nuclei stained by DAPI, scale bar = 50 μm. (D) Mean fluorescence intensity of p-Smad2/3 in fibroblasts treated with different treatments, with **p < 0.01 vs. the indicated group.** Fig. S6**. The effect of ZNF416 siRNA-loaded liposomes in vivo. (A) The body weight of the mice in the control and the ZNF416 siRNA-loaded liposomes group. (B-F) The levels of alanine aminotransferase/pyruvate transaminase (ALT/GPT) and aspartate aminotransferase (AST), the serum levels of creatinine (Scr), blood urea nitrogen (BUN) and creatine kinase-MB (CK-MB) in the mice serum. (G) The H&E staining of the different organs including heart, liver, kidney and lung in the control and the ZNF416 siRNA-loaded liposomes group.** Fig. S7**. Administration of ZNF416 siRNA-loaded liposomes attenuates BLM-induced mouse fibrosis. (A) Experimental design. 8-week-old C57BL/6 mice were injected intratracheally with BLM (6 mg/kg) or DMSO. ZNF416 siRNA-loaded or scrambled liposomes (1mg/kg) were injected into the anesthetized animals by tail vein on days 7 and 14 following BLM administration. (B) qRT-PCR analysis of relative expression of ZNF416, α-SMA and Collagen I in the indicated groups, with **p < 0.01 vs. the control group and #p < 0.05 vs. the scramble liposomes group. (C) Representative hematoxylin and eosin (H&E) and Masson’s trichrome staining and Sirius red of lung sections from the indicated groups. scale bar = 100 μm. (D) Immunofluorescence staining with Collagen I and α-SMA in mouse lung slices from the different groups. scale bar = 100 μm. (E-F) Mean fluorescence intensity of Collagen I and α-SMA in lung slices from the different groups, with **p < 0.01 vs. control group and #p < 0.05 vs. the scramble liposomes group. (G) Protein levels of fibrotic markers, ZNF416, Smad2/3 and p-Smad2/3 in the different groups. (H) Hydroxyproline levels in lungs of C57/BL6 mice from the different groups, with **p < 0.01 vs. control group and #p < 0.05 vs. the scramble liposomes group.** Fig. S8**. Combination of ZNF416 siRNA-loaded liposomes and SB431542 exerts a synergic effect on BLM‐induced pulmonary fibrosis. (A) Strategy for co-administration of ZNF416 siRNA-loaded liposomes and SB431542 in the BLM-induced pulmonary fibrosis mouse model. (B) Quantification of hydroxyproline contents in mice after BLM challenge for the indicated group, with **p < 0.01 vs. the indicated group. (C) H&E staining, Masson staining, and Sirius red staining were performed to measure the severity of lung fibrosis. (D-E) Western blot and qRT‐PCR analysis of fibrotic markers and ZNF416 protein and mRNA level in mouse lung tissues on saline, BLM, BLM + siZNF416 liposomes, BLM + siZNF416 liposomes + SB431542 and BLM + scramble liposomes + DMSO group, with **p < 0.01 vs. the indicated group.** Table S1**. The specific details of the primary antibodies include western blot (WB), immunofluorescence (IF), and immunohistochemistry (IHC).** Table S2**. Primer Sequence for RT-qPCR.

## Abbreviations

ECM: Extracellular matrix
TGF-β1: Transforming growth factor-beta 1
BLM: Bleomycin
YAP: Yes-associated protein
MRTF-A: Myocardin-related transcription factor A
SRF: Serum response factor
IPF: Idiopathic pulmonary fibrosis
H&E: Hematoxylin and eosin
IHC: Immunohistochemical
TEM: Transmission electron microscopy
IVIS: In vivo imaging system
ALT/GPT: Alanine aminotransferase/pyruvate transaminase
AST: Aspartate aminotransferase
Scr: The serum levels of creatinine
BUN: Blood urea nitrogen
CK-MB: Creatine kinase-MB
ROCK: Rho-associated coiled-coil forming kinase
RNAi: RNA interference
