# Supplementary material for: Targeted delivery of ZNF416 siRNA-loaded liposomes attenuates experimental pulmonary fibrosis
Source: J Transl Med. 2022 Nov 12;20:523. doi: 10.1186/s12967-022-03740-w (PMC9652794; doi:10.1186/s12967-022-03740-w)
Supplement: Supplementary file 1 — Additional file 1: Fig. S1. ZNF416 expression is increased in mechanics-induced fibroblasts and fibrotic lung tissues. (A) mRNA levels of ZNF416 were determined by qRT-PCR in the mouse lung tissues of the control, silicosis and BLM group, with **p < 0.01 and *p < 0.05 vs. the control group. (B) Western blot and densitometric analysis of ZNF416 and fibrotic markers including Fibronectin, Collagen I and α-SMA expression in mouse lungs of the control (n = 3) and BLM (n = 3) group, with **p < 0.01 vs. the control group. (C-D) Immunofluorescence analysis of α-SMA and ZNF416 in mouse lung slices from the different groups. scale bar = 100 μm. Fig. S2. Increasing matrix stiffness induced fibroblast activation via ZNF416. (A-B) Mean fluorescence intensity of α-SMA and Collagen I in NIH/3T3 cells culture on 1 or 60 kappa collagen-coated hydrogels coverslips, with **p < 0.01 vs. 1 kappa group. (C) Immunofluorescence analysis of Actin in NIH/3T3 cells culture on 1 or 60 kappa collagen-coated hydrogels coverslips. Actin stained red, Nuclei were stained by DAPI, scale bar = 25 μm. (D) Western blot showed the silencing efficacy of siRNAs against ZNF416 in NIH/3T3 cells culture on 1 or 60 kappa collagen-coated hydrogels coverslips plus with ZNF416 #1-#3 siRNA. (E) qRT-PCR analysis of relative expression of fibrotic markers after ZNF416 knockdown culture on 1 kappa collagen-coated hydrogels coverslips, with **p < 0.01 vs. the 1 kappa group. Fig. S3. Gain- of function ZNF416 facilitates fibroblast differentiation, proliferation and contraction. (A) qRT-PCR analysis of relative expression of ZNF416 in NIH/3T3 cells treated with ZNF416 plasmid, with **p < 0.01 vs. control group. (B) The densitometric analysis demonstrated a dramatically increased synthesis of fibrosis-related proteins after treatment of ZNF416 plasmid in MRC-5 cells, with **p < 0.01 vs. control group and #p < 0.05 vs. ZNF416 plasmid group. (C) Western blot demonstrated a dramatically increased expression of fibrosis-re [file 12967_2022_3740_MOESM1_ESM.docx]

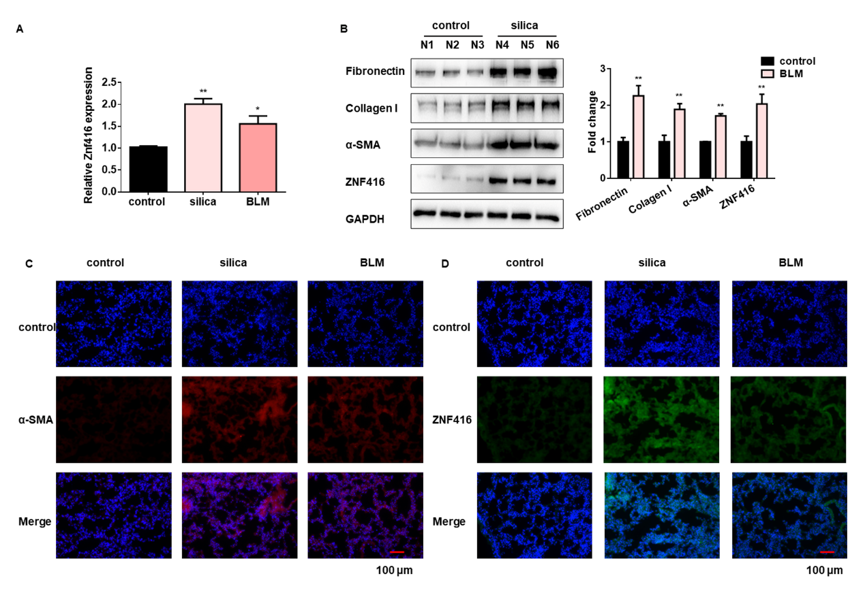
**Fig. S1.** ZNF416 expression is increased in mechanics-induced fibroblasts and fibrotic lung tissues. (A) mRNA levels of ZNF416 were determined by qRT-PCR in the mouse lung tissues of the control, silicosis and BLM group, with ^**^*p* < 0.01 and ^*^*p* < 0.05 vs. the control group. (B) Western blot and densitometric analysis of ZNF416 and fibrotic markers including Fibronectin, Collagen I and α-SMA expression in mouse lungs of the control (n = 3) and BLM (n = 3) group, with ^**^*p* < 0.01 vs. the control group. (C-D) Immunofluorescence analysis of α-SMA and ZNF416 in mouse lung slices from the different groups. scale bar = 100 μm.

**Fig. S2.** Increasing matrix stiffness induced fibroblast activation via ZNF416. (A-B) Mean fluorescence intensity of α-SMA and Collagen I in NIH/3T3 cells culture on 1 or 60 kappa collagen-coated hydrogels coverslips, with ^**^*p* < 0.01 vs. 1 kappa group. (C) Immunofluorescence analysis of Actin in NIH/3T3 cells culture on 1 or 60 kappa collagen-coated hydrogels coverslips. Actin stained red, Nuclei were stained by DAPI, scale bar = 25 μm. (D) Western blot showed the silencing efficacy of siRNAs against ZNF416 in NIH/3T3 cells culture on 1 or 60 kappa collagen-coated hydrogels coverslips plus with ZNF416 #1-#3 siRNA. (E) qRT-PCR analysis of relative expression of fibrotic markers after ZNF416 knockdown culture on 1 kappa collagen-coated hydrogels coverslips, with ^**^*p* < 0.01 vs. the 1 kappa group.


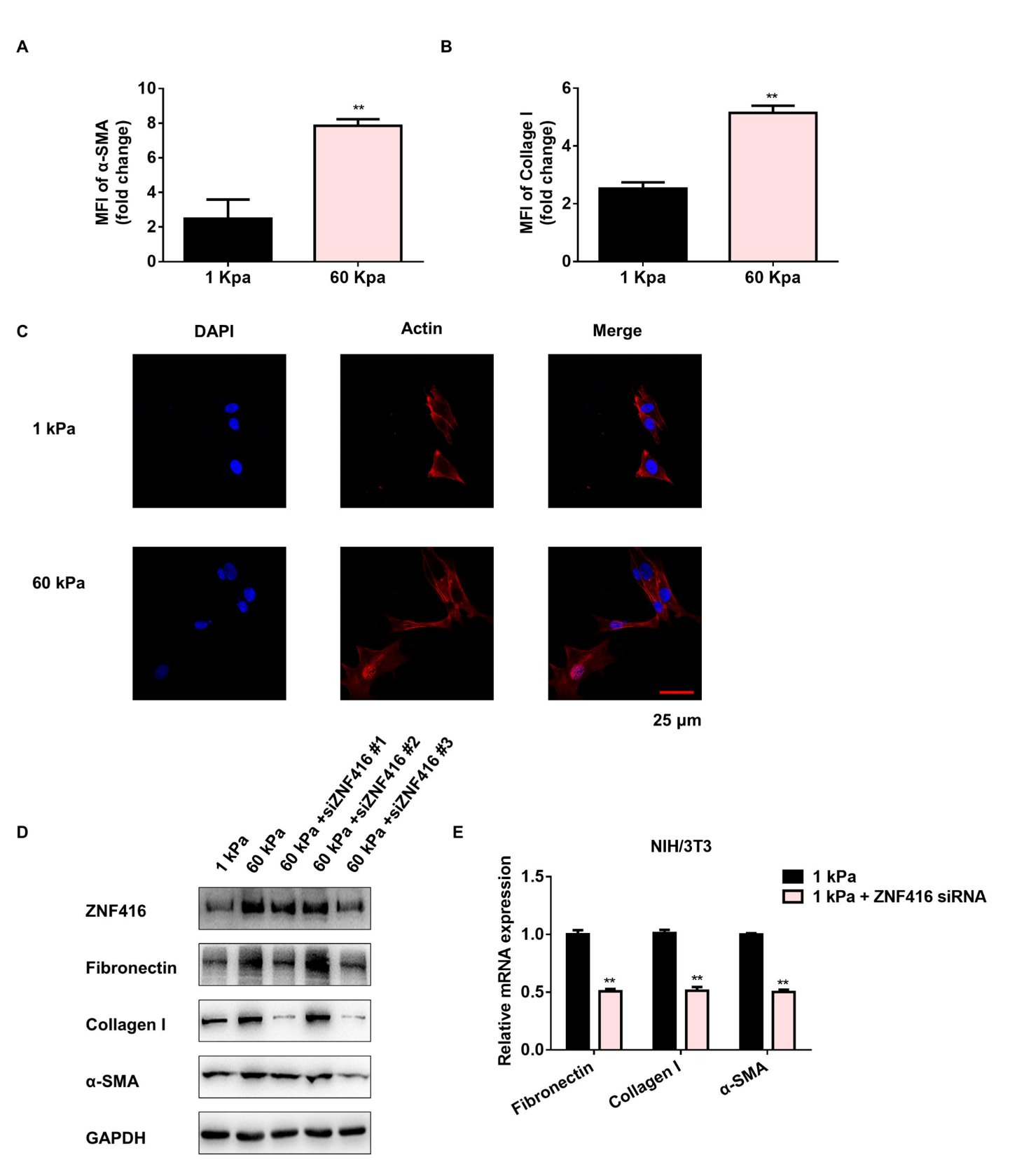


**Fig. S3.** Gain- of function ZNF416 facilitates fibroblast differentiation, proliferation and contraction. (A) qRT-PCR analysis of relative expression of ZNF416 in NIH/3T3 cells treated with ZNF416 plasmid, with ^**^*p* < 0.01 vs. control group. (B) The densitometric analysis demonstrated a dramatically increased synthesis of fibrosis-related proteins after treatment of ZNF416 plasmid in MRC-5 cells, with ^**^*p* < 0.01 vs. control group and ^#^*p* < 0.05 vs. ZNF416 plasmid group. (C) Western blot demonstrated a dramatically increased expression of fibrosis-related protein after treatment of ZNF416 plasmid in NIH/3T3 cells. (D-E) Immunofluorescence analysis of Fibronectin and Collagen I after treatment of ZNF416 plasmid in NIH/3T3 cells, Fibronectin stained red, Collagen stained green, Nuclei were stained by DAPI, scale bar = 50 μm. (F) Mean fluorescence intensity of Collagen I in MRC-5 cells treated with ZNF416 plasmid or ZNF416 plasmid NC, with ^**^*p* < 0.01 vs. the control group and ^#^*p* < 0.05 vs. the ZNF416 plasmid group. (G) Cell proliferation of MRC-5 cells transfected with ZNF416 plasmid or ZNF416 plasmid NC, with ^**^*p* < 0.01 vs. the control group and ^#^*p* < 0.05 vs. the ZNF416 plasmid group.


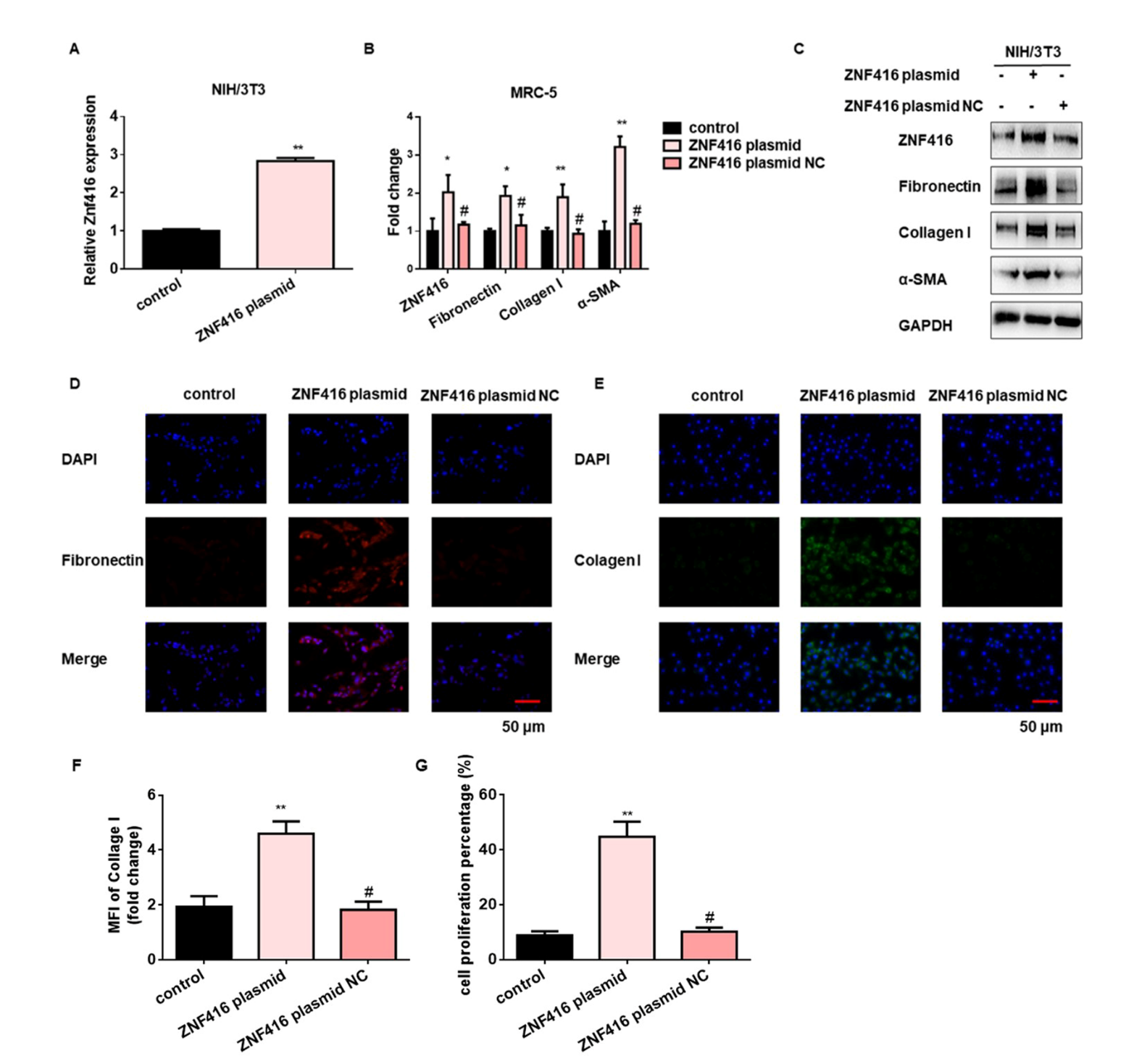
**Fig. S4.** Loss- of function ZNF416 attenuates fibroblast differentiation, proliferation and contraction. (A) MTT assays were performed to evaluate cell proliferative ability in fibroblasts. (B) CCK8 assays were performed to evaluate the effect of ZNF416 siRNA on cells, with ^**^*p* < 0.01 vs. the control group. (C) qRT-PCR analysis of relative expression of ZNF416 in NIH/3T3 cells treated with ZNF416 siRNA, with ^**^*p* < 0.01 vs. control group. (D) Western blots analysis demonstrated a dramatically decreased expression of fibrosis-related proteins after treatment of ZNF416 siRNA in NIH/3T3 cells. (E) qRT-PCR demonstrated a dramatically decreased expression of fibrosis-related genes after treatment of ZNF416 siRNA in NIH/3T3 cells, with ^**^*p* < 0.01 vs. control group. (F-G) Immunofluorescence analysis of Fibronectin and Collagen I after being treated with ZNF416 siRNA in NIH/3T3 cells, Fibronectin stained red, Collagen stained green, Nuclei were stained by DAPI, scale bar = 50 μm. (H) Mean fluorescence intensity of Collagen I in MRC-5 cells treated with different treatments, with ^**^*p* < 0.01 vs. the control group and ^#^*p* < 0.05 vs. the TGF-β1 group. (I) Cell proliferation of MRC-5 cells transfected with ZNF416 siRNA, with ^**^*p* < 0.01 vs. the control group and ^#^*p* < 0.05 vs. the the TGF-β1 group.


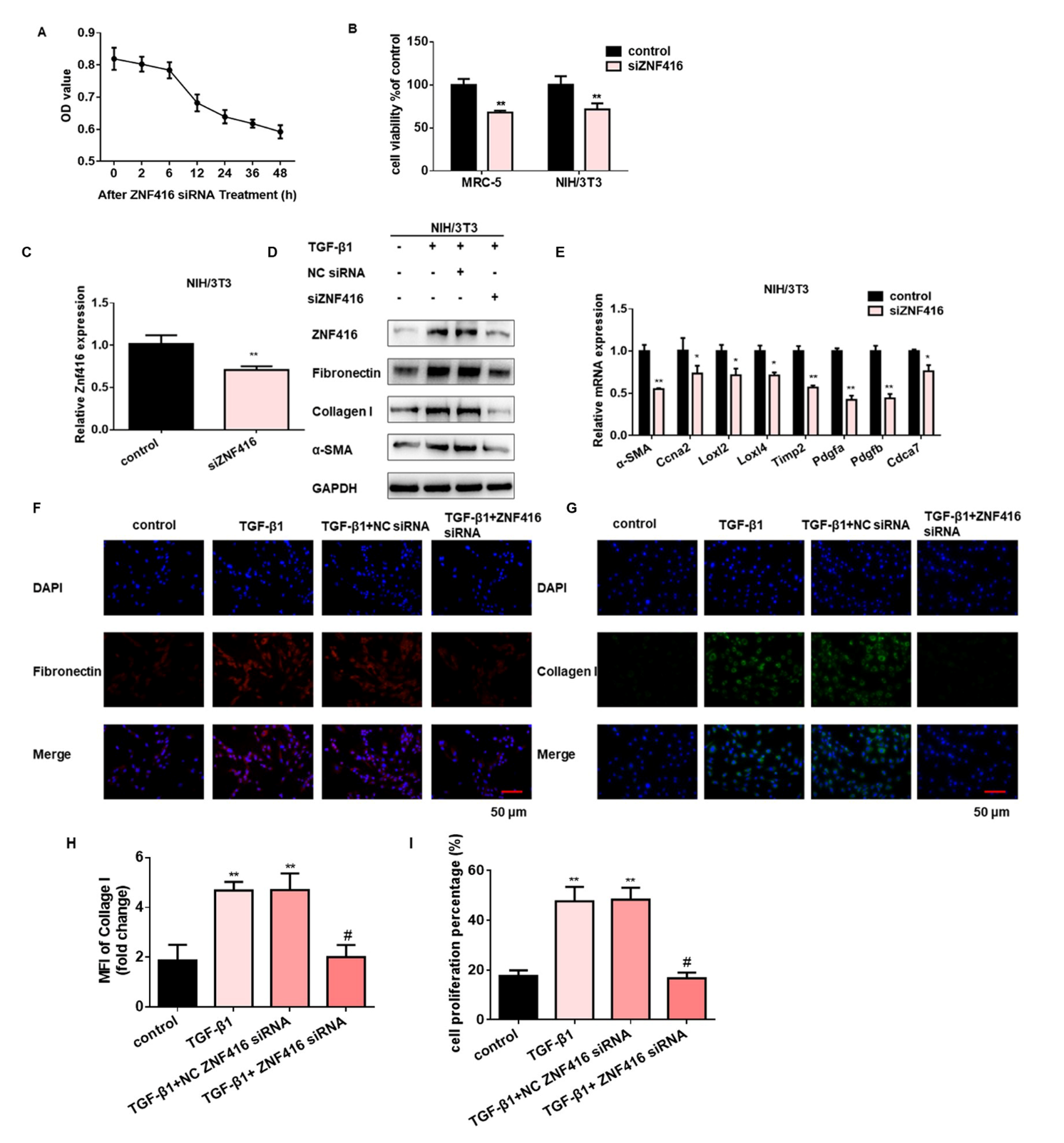
**Fig. S5.** ZNF416 regulates the activation of fibroblasts by promoting the nuclear accumulation of p-Smad2/3. (A) mRNA levels of Smad2 and Smad3 were determined by qRT-PCR in NIH/3T3 cells treated with ZNF416 plasmid or ZNF416 siRNA. (B) Protein levels of Smad2/3 and p-Smad2/3 were determined by western blot after ZNF416 knockdown. (C) Immunofluorescence analysis of p-Smad2/3 after treatment with different treatments in MRC-5 cells, p-Smad2/3 stained red, ZNF416 stained green, Nuclei stained by DAPI, scale bar = 50 μm. (D) Mean fluorescence intensity of p-Smad2/3 in fibroblasts treated with different treatments,
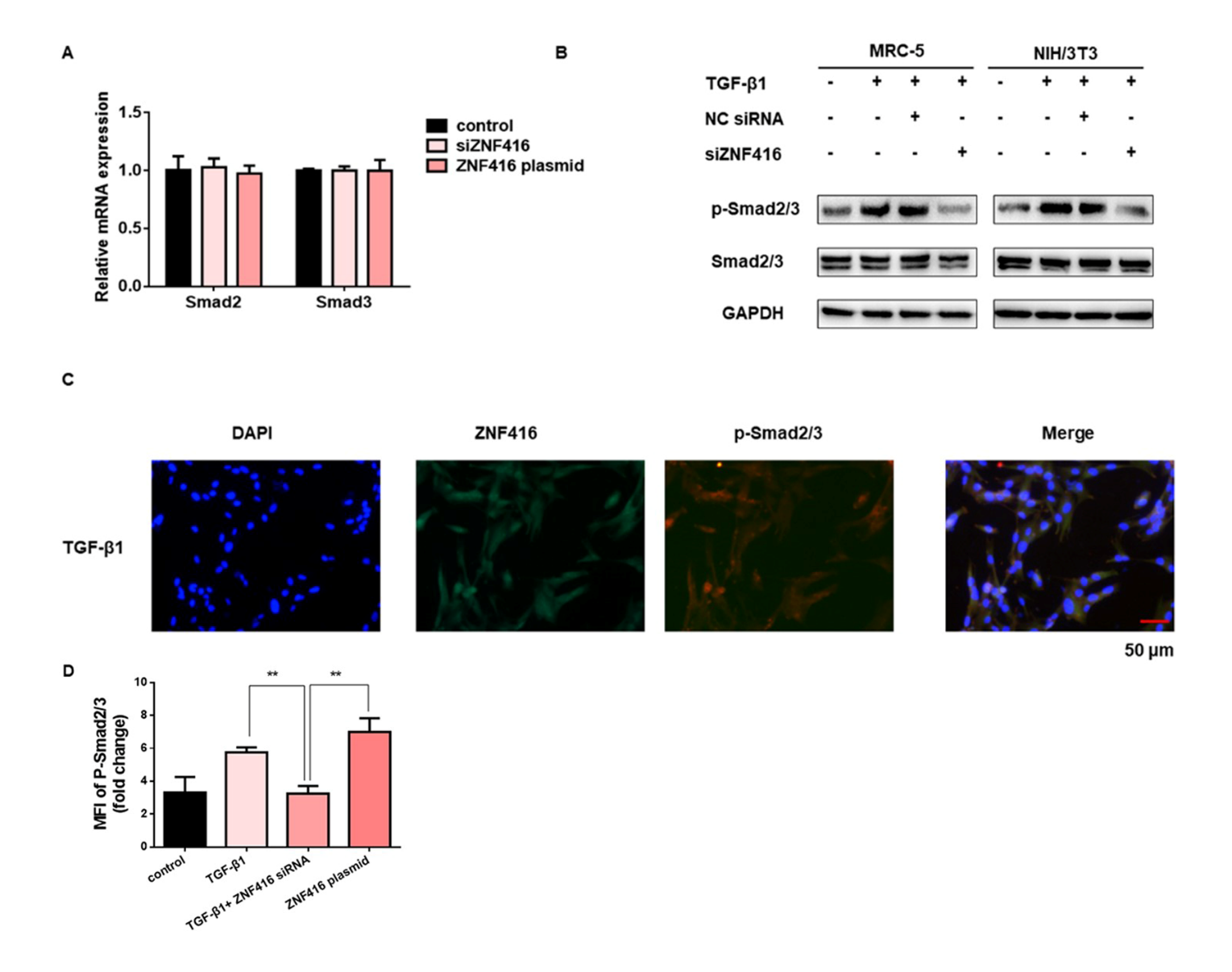
with ^**^*p* < 0.01 vs. the indicated group.

**Fig. S6. The effect of ZNF416 siRNA-loaded liposomes in vivo.** (A) The body weight of the mice in the control and the ZNF416 siRNA-loaded liposomes group. (B-F) The levels of alanine aminotransferase/pyruvate transaminase (ALT/GPT) and aspartate aminotransferase (AST), the serum levels of creatinine (Scr), blood urea nitrogen (BUN) and creatine kinase-MB (CK-MB) in the mice serum. (G) The H&E staining of the different organs including heart, liver, kidney and lung in the
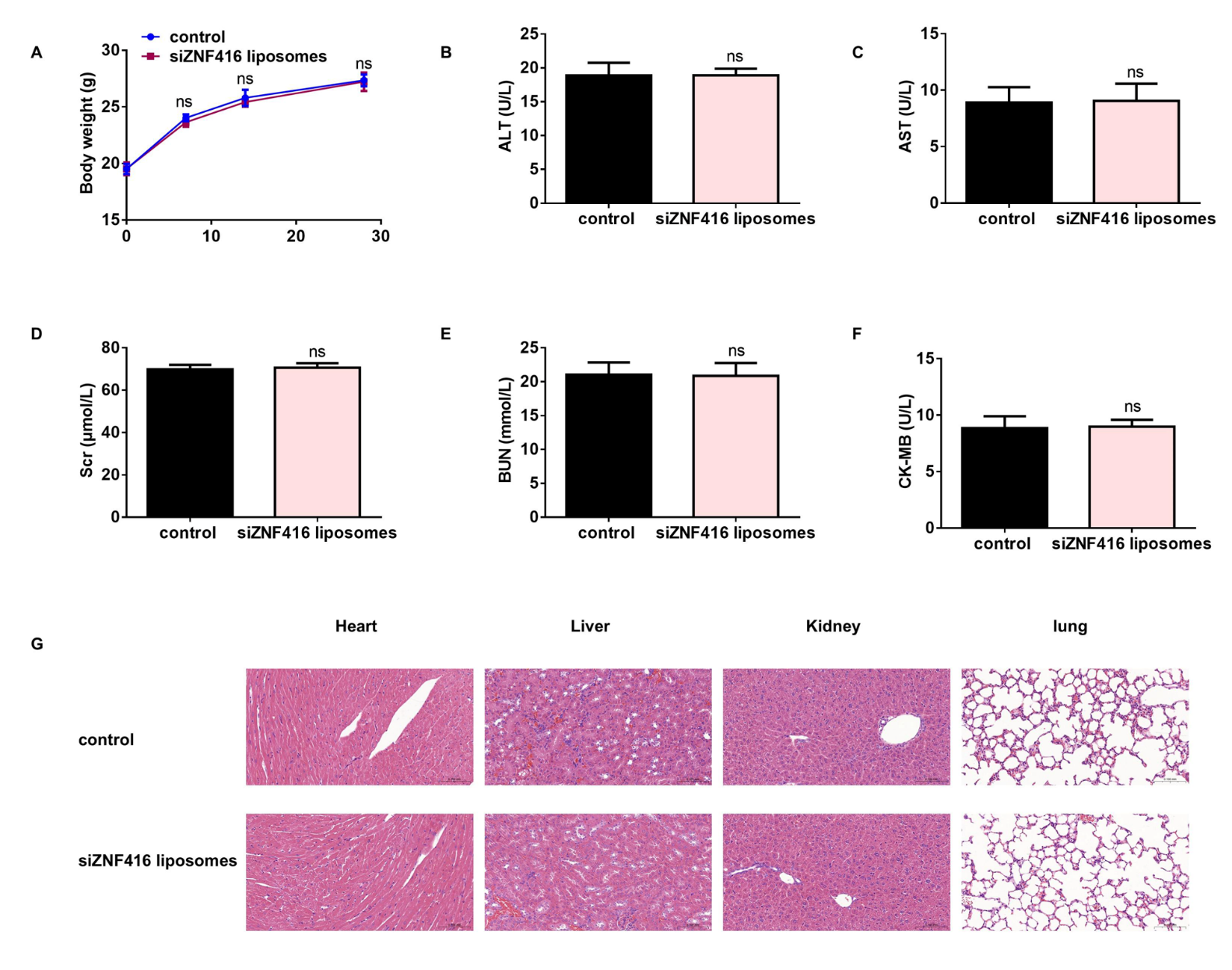
control and the ZNF416 siRNA-loaded liposomes group.

**Fig. S7.** Administration of ZNF416 siRNA-loaded liposomes attenuates BLM-induced mouse fibrosis. (A) Experimental design. 8-week-old C57BL/6 mice were injected intratracheally with BLM (6 mg/kg) or DMSO. ZNF416 siRNA-loaded or scrambled liposomes (1mg/kg) were injected into the anesthetized animals by tail vein on days 7 and 14 following BLM administration. (B) qRT-PCR analysis of relative expression of ZNF416, α-SMA and Collagen I in the indicated groups, with ^**^*p* < 0.01 vs. the control group and ^#^*p* < 0.05 vs. the scramble liposomes group. (C) Representative hematoxylin and eosin (H&E) and Masson’s trichrome staining and Sirius red of lung sections from the indicated groups. scale bar = 100 μm. (D) Immunofluorescence staining with Collagen I and α-SMA in mouse lung slices from the different groups. scale bar = 100 μm. (E-F) Mean fluorescence intensity of Collagen I and α-SMA in lung slices from the different groups, with ^**^*p* < 0.01 vs. control group and ^#^*p* < 0.05 vs. the scramble liposomes group. (G) Protein levels of fibrotic markers, ZNF416, Smad2/3 and p-Smad2/3 in the different groups. (H) Hydroxyproline levels in lungs of C57/BL6 mice from the different groups, with ^**^*p* < 0.01 vs. control group and ^#^*p* < 0.05 vs. the scramble liposomes group.


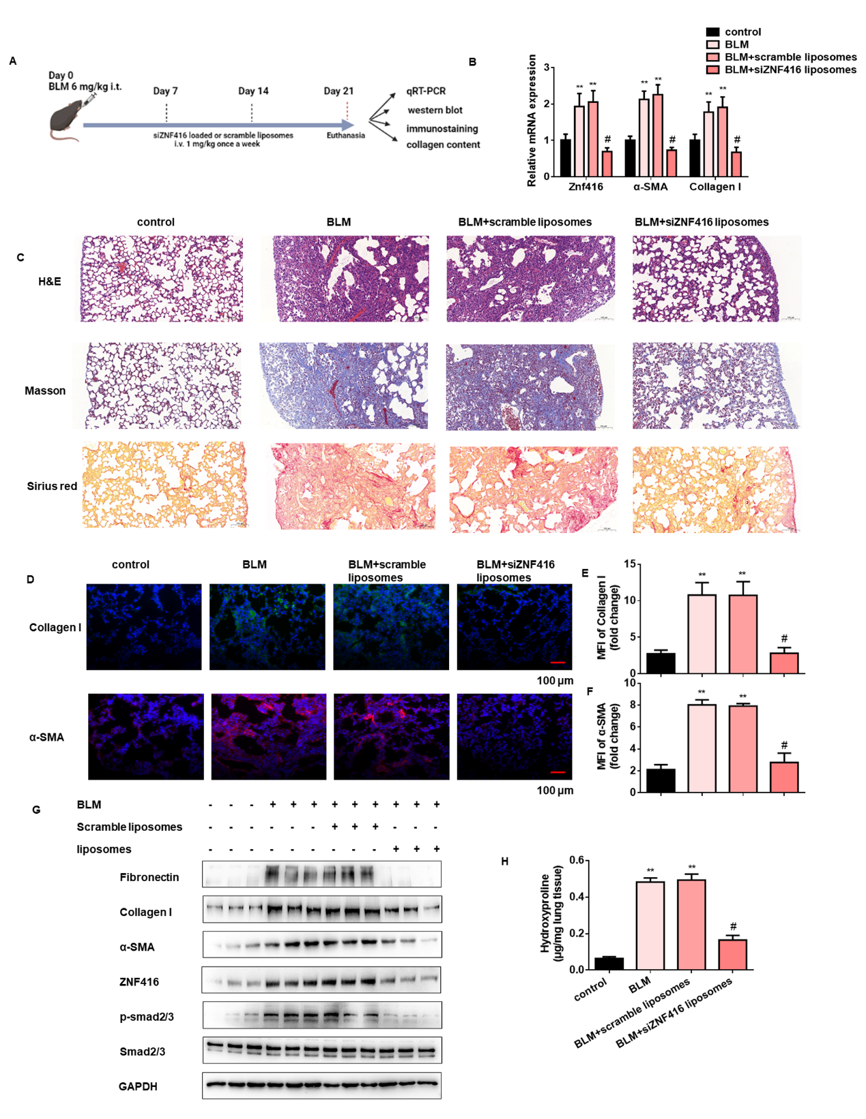
**Fig. S8.** Combination of ZNF416 siRNA-loaded liposomes and SB431542 exerts a synergic effect on BLM‐induced pulmonary fibrosis. (A) Strategy for co-administration of ZNF416 siRNA-loaded liposomes and SB431542 in the BLM-induced pulmonary fibrosis mouse model. (B) Quantification of hydroxyproline contents in mice after BLM challenge for the indicated group, with ^**^*p* < 0.01 vs. the indicated group. (C) H&E staining, Masson staining, and Sirius red staining were performed to measure the severity of lung fibrosis. (D-E) Western blot and qRT‐PCR analysis of fibrotic markers and ZNF416 protein and mRNA level in mouse lung tissues on saline, BLM, BLM + siZNF416 liposomes, BLM + siZNF416 liposomes + SB431542 and BLM + scramble liposomes + DMSO group, with ^**^*p* <
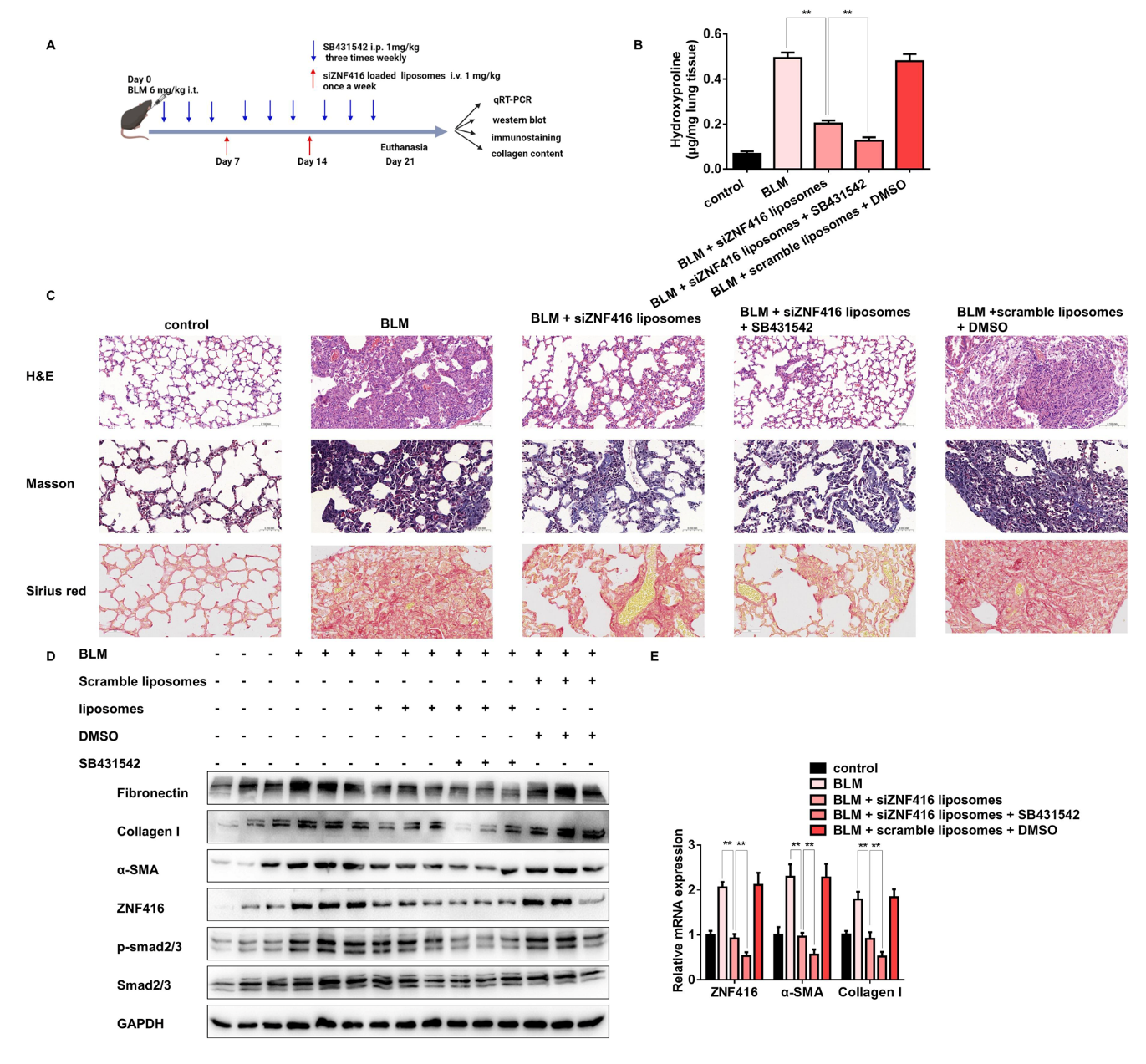
0.01 vs. the indicated group.

Supplementary Table 1

| **Antibody** | **Dilution ratio** |
| --- | --- |
| GAPDH (ABclonal, AC002) | 1:1000 for WB |
| Fibronectin (Abcam, ab45688) | 1:1000 for WB; 1:400 for IF |
| Collagen I (ABclonal, A1352) | 1:1000 for WB; 1:400 for IF |
| α-SMA (Abcam, ab32575) | 1:1000 for WB; 1:400 for IF |
| ZNF416 (Invitrogen, PA5-100791) | 1:1000 for WB; 1:400 for IF; 1:200 for IHC |
| p-Smad2/3 (Elabscience, E-AB-21040) | 1:1000 for WB; 1:400 for IF; |
| Smad2/3 (Cell Signaling, #8685) | 1:1000 for WB |

The specific details of the primary antibodies include western blot (WB), immunofluorescence (IF), and immunohistochemistry (IHC).

Supplementary Table 2

| Gene Name | Forward primer (5'-3') | Reverse primer (3'-5') |
| --- | --- | --- |
| ZNF416 (Homo) | TAACTGCGCTGGTTTGTTGG | GCAAATCGGTCAGGTGCAAAAT |
| α-SMA (Homo) | CTATGAGGGCTATGCCTTGCC | GCTCAGCAGTAGTAACGAAGGA |
| CCNA2 (Homo) | CGCTGGCGGTACTGAAGTC | GAGGAACGGTGACATGCTCAT |
| LOXL2 (Homo) | GGGTGGAGGTGTACTATGATGG | CTTGCCGTAGGAGGAGCTG |
| LOXL4 (Homo) | CTGGGCACCACTAAGCTCC | CTCCTGGATAGCAAAGTTGTCAT |
| TIMP2 (Homo) | AAGCGGTCAGTGAGAAGGAAG | GGGGCCGTGTAGATAAACTCTAT |
| PDGFA (Homo) | GCAAGACCAGGACGGTCATTT | GGCACTTGACACTGCTCGT |
| PDGFB (Homo) | CTCGATCCGCTCCTTTGATGA | CGTTGGTGCGGTCTATGAG |
| CDCA7 (Homo) | GGTCCCTTGACGCTCTACC | TGGGCGAATTATATGCGGAAG |
| GAPDH (Homo) | CCTTCCGTGTCCCCACT | GCCTGCTTCACCACCTTC |
| Znf416 (Mus) | ACAGGGCTGTGTAACCTTTGA | CTGCTTCAGACCAATGAGCCA |
| α-SMA (Mus) | GGCACCACTGAACCCTAAGG | ACAATACCAGTTGTACGTCCAGA |
| Ccna2 (Mus) | AAGAGAATGTCAACCCCGAAAAA | ACCCGTCGAGTCTTGAGCTT |
| Loxl2 (Mus) | AGCCTAGCACAGTACGAGGG | TGGATCTTGACCACATCGGAG |
| Loxl4 (Mus) | GCCAACGGACAGACCAGAG | CCAGGTCAAGGCTGACTCAAA |
| Timp2 (Mus) | TCAGAGCCAAAGCAGTGAGC | GCCGTGTAGATAAACTCGATGTC |
| Pdgfa (Mus) | TGGCTCGAAGTCAGATCCACA | TTCTCGGGCACATGGTTAATG |
| Pdgfb (Mus) | TCCGGCTGCTGCAATAACC | GGCTTCTTTCGCACAATCTCAAT |
| Cdca7 (Mus) | GCCTCTCAGGGTAGCAATGAA | CAGAGTGGGAATCGTTTGCAG |
| Gapdh (Mus) | TGTTTCCTCGTCCCGTAGA | ATCTCCACTTTGCCACTGC |

Primer Sequence for RT-qPCR
